# Epigenetic targets of Janus kinase inhibitors are linked to genetic risks of rheumatoid arthritis

**DOI:** 10.1186/s41232-024-00337-2

**Published:** 2024-06-04

**Authors:** Haruka Tsuchiya, Mineto Ota, Haruka Takahashi, Hiroaki Hatano, Megumi Ogawa, Sotaro Nakajima, Risa Yoshihara, Tomohisa Okamura, Shuji Sumitomo, Keishi Fujio

**Affiliations:** 1 Department of Allergy and Rheumatology, Graduate School of Medicine, The University of Tokyo, 7-3-1 Hongo, Bunkyo-Ku, Tokyo, 113-0033 Japan; 2 Department of Functional Genomics and Immunological Diseases, Graduate School of Medicine, The University of Tokyo, 7-3-1 Hongo, Bunkyo-Ku, Tokyo, 113-0033 Japan

**Keywords:** Janus kinase inhibitor, Rheumatoid arthritis, Synovial fibroblasts, Tumor necrosis factor-α inhibitor

## Abstract

**Background:**

Current strategies that target cytokines (e.g., tumor necrosis factor (TNF)-α), or signaling molecules (e.g., Janus kinase (JAK)) have advanced the management for allergies and autoimmune diseases. Nevertheless, the molecular mechanism that underpins its clinical efficacy have largely remained elusive, especially in the local tissue environment. Here, we aimed to identify the genetic, epigenetic, and immunological targets of JAK inhibitors (JAKis), focusing on their effects on synovial fibroblasts (SFs), the major local effectors associated with destructive joint inflammation in rheumatoid arthritis (RA).

**Methods:**

SFs were activated by cytokines related to inflammation in RA, and were treated with three types of JAKis or a TNF-α inhibitor (TNFi). Dynamic changes in transcriptome and chromatin accessibility were profiled across samples to identify drug targets. Furthermore, the putative targets were validated using luciferase assays and clustered regularly interspaced short palindromic repeat (CRISPR)-based genome editing.

**Results:**

We found that both JAKis and the TNFi targeted the inflammatory module including *IL6*. Conversely, specific gene signatures that were preferentially inhibited by either of the drug classes were identified. Strikingly, RA risk enhancers for *CD40* and *TRAF1* were distinctively regulated by JAKis and the TNFi. We performed luciferase assays and CRISPR-based genome editing, and successfully fine-mapped the single causal variants in these loci, rs6074022-*CD40* and rs7021049-*TRAF1.*

**Conclusions:**

JAKis and the TNFi had a direct impact on different RA risk enhancers, and we identified nucleotide-resolution targets for both drugs. Distinctive targets of clinically effective drugs could be useful for tailoring the application of these drugs and future design of more efficient treatment strategies.

**Supplementary Information:**

The online version contains supplementary material available at 10.1186/s41232-024-00337-2.

## Background

Rheumatoid arthritis (RA) causes persistent synovitis leading to disabling joint destruction. In RA pathogenesis, various molecules produced by immune cells (e.g., T cells, B cells, and monocytes) and mesenchymal cells are dysregulated through the influence of genetic predisposition and environmental factors [1]. Notably, synovial fibroblasts (SFs), the most abundant resident mesenchymal cells in the synovium, are major local effectors in the initiation and perpetuation of destructive joint inflammation by producing a variety of pathogenic molecules including interleukin (IL)-6 [2].

Current treatment strategies that target cytokines (e.g., tumor necrosis factor (TNF)-α, IL-6), cell surface proteins (e.g., CD20, CD80/86), or signaling molecules (e.g., Janus kinase (JAK)) have brought a paradigm shift in RA treatment [3]. In particular, JAK inhibitors (JAKis) have emerged as orally available low molecular weight products. The JAK family comprises JAK1, JAK2, JAK3, and tyrosine kinase 2 (TYK2). The JAK pair involved in signal transduction differs depending on the type of humoral factor that it binds [4, 5]. Following activation and transphosphorylation of JAKs, the signal transducer and activator of transcription (STAT) dimerizes and regulates transcription associated with inflammation, hematopoiesis, and immune homeostasis in the nucleus. The potent and preferential inhibition of JAK1 of the present JAKis is regarded to be largely responsible for the efficacy in RA [6, 7], besides varying levels of biochemical selectivity for other JAK isoforms [8].

So far, many JAKis have been used in randomized controlled trials (RCTs) for RA (e.g., tofacitinib, baricitinib, and upadacitinib). Overall, there are potential class benefits of JAKis (e.g., rapid onset of action and a low risk of immunogenicity) as monotherapy or in combination with background conventional synthetic disease-modifying anti-rheumatic drugs (DMARDs) [9–21], and in specific patient groups, numerical and statistical advantages over a TNF-α inhibitor (TNFi; adalimumab) have been reported in head-to-head trials [22–24]. Nevertheless, the predisposing genetic, epigenetic, and immunological properties that underpin the clinical efficacy of JAKis have largely remained elusive, especially compared to TNFis.

Here, we assessed points of action of JAKis (tofacitinib, baricitinib, and upadacitinib) and a TNFi (adalimumab) on activated SFs by integrative methods to analyze genomics, transcriptomics, and epigenomics. Analyses of SFs treated with these therapeutic agents identified gene signatures and modifications of enhancer structures associated with RA heritability, which are characteristically provided by each drug class. Moreover, we successfully fine-mapped causal single-nucleotide polymorphisms (SNPs), rs6074022-*CD40* and rs7021049-*TRAF1*, located in the respective target regions of JAKis and the TNFi.

## Methods

### Study design

The overall objectives of this study were to explore the immunological properties underpinning the efficacy of Janus kinase inhibitors (JAKis) and a tumor necrosis factor (TNF)-α inhibitor (TNFi) on the inflammatory phenotype of rheumatoid arthritis (RA) synovial fibroblasts (SFs). First, primary SFs from RA patients (*n* = 6) were stimulated with a combination of three disease-relevant cytokines (TNF-α, interleukin (IL)-1β, and interferon (IFN)-γ), and treated with three types of JAKis (tofacitinib, baricitinib, or upadacitinib) or a TNFi (adalimumab). We quantified transcript expression by RNA sequencing and gained information on open chromatin structure by ATAC sequencing 24 h and 7 days after treatment. Next, weighted gene co-expression network analysis (WGCNA) was performed, and we examined the relationship between temporal perturbations and therapeutic responsiveness in each module. In addition, we constructed an enhancer-gene map using an activity-by-contact (ABC) model to elucidate the regulatory mechanisms of gene expression in activated and quiescent SFs. In this analytical pipeline, we combined the information of open chromatin regions (defined with ATAC sequencing), active regulatory regions (defined with H3K27ac ChIP sequencing analysis), and the 3D genome architectures (chromatin loops detected by Hi-C analysis). Focusing on the ABC enhancer regions associated with RA heritability that were directly modified by therapeutic agents, we examined causal single nucleotide polymorphisms (SNPs) for the *TRAF1* and *CD40* locus using a luciferase assay and clustered regularly interspaced short palindromic repeat (CRISPR)-based genome editing. Finally, we conducted stratified linkage disequilibrium score regression (S-LDSC) analysis to assess the enrichment of heritability of various immune-mediated diseases including RA in target gene sets of each therapeutic agent.

### Cell lines and cell culture

Primary RA SFs were purchased from Articular Engineering. The clinical background of the providers of RA SFs is summarized in Supplementary Table 1. The Riken cell bank provided the MH7A cells. The HT-1080 cells were obtained from the Japanese Collection of Research Bioresources Cell Bank, National Institutes of Biomedical Innovation, Health and Nutrition. RA SFs and the MH7A cells were cultured in Dulbecco’s modified Eagle’s medium (DMEM; Invitrogen) supplemented with 10% fetal bovine serum (FBS; BioWest), 100 µg/mL L-glutamine, 100 U/mL penicillin, and 100 µg/mL streptomycin (all from Invitrogen) at 37℃, in 5% CO_2_. The HT-1080 cells were cultured in DMEM (Gibco) supplemented with 10% FBS (BioWest), 0.1 mM MEM non-essential amino acids solution (Gibco), 100 µg/mL L-glutamine, 100 U/mL penicillin, and 100 µg/mL streptomycin (all from Invitrogen) at 37℃, in 5% CO2. The medium was changed once every 3 days and was subcultured with 0.2% trypsin–EDTA (Invitrogen) when cells reached 80–90% confluence. RA SFs from passages 2 or 3 were used for all experiments.

### Cytokine stimulation and drug treatment of RA SFs

Primary RA SFs (*n* = 6) were seeded with DMEM (10% FBS, 100 µg/mL L-glutamine, 100 U/mL penicillin, 100 µg/mL streptomycin) into a 24-well flat-bottom plate (Corning) with a density of 2 × 10^4^ cells per well and incubated at 37 °C, in 5% CO_2_. After 12 h, a mixture of three cytokines (200 U/mL IFN-γ, 10 ng/mL TNF-α, and 10 ng/mL IL-1β (all from PeproTech)) that simulated synergistic inflammation in arthritic joints was added. The cells were stimulated for an additional 12 h at 37 °C, in 5% CO_2_, and one of the following inhibitors was added: 20 µg/ml adalimumab (AbbVie), 4 µM tofacitinib (Selleck), 4 µM baricitinib (Selleck), or 4 µM upadacitinib (AbbVie). The medium containing each drug and cytokine was replaced 72 h after the first drug addition. Drug-treated RA SFs were harvested after 24 h (acute phase) and 7 days (chronic phase) for RNA and ATAC sequencing.

### RNA sequencing

Total RNA from RA SFs was isolated using the RNeasy micro kit (Qiagen). Libraries for RNA sequencing were prepared using the TruSeq Stranded mRNA Library Prep Kit (Illumina). RNA sequencing was carried out on an Illumina NovaSeq 6000 (read length of 150 bp, paired end).

### Bioinformatic analysis of RNA sequencing data

From RNA sequencing reads, adapters were trimmed with cutadapt (version 1.16) (key resources information) and low-quality reads were trimmed using the FASTX-Toolkit (version 0.0.14) (key resources information). Subsequently, reads were aligned to the GRCh38 reference genome using STAR (version 2.5.3) (key resources information) in two-pass mode with Gencode version 27 annotations (key resources information). Only uniquely mapped read pairs were used for analysis. Expression was quantified using HTSeq (version 0.11.2) (key resources information). Differentially expressed genes (DEGs) were identified using dream software implemented in the variancePartition package in R (key resources information), with sample donors treated as random effect variables in a linear mixed model.

### ATAC sequencing

Libraries for ATAC sequencing were prepared as per the Omni-ATAC sequencing protocol [25]. Briefly, 50,000 RA SFs were lysed in 10 mM tris–HCl (pH 7.4), 10 mM NaCl, 3 mM MgCl_2_, and 0.1% Tween 20. The nuclear pellet was then subjected to a transposition reaction using the Tagment DNA TDE1 Enzyme and Buffer Kit (Illumina) in the presence of 0.01% digitonin and 0.1% Tween 20 at 37 °C for 30 min and cleaned up with DNA Clean and Concentrator-5 Kit (Zymo). For amplification of transposed DNA, quantitative PCR was performed using NEBNext High-Fidelity 2 × PCR Master Mix (NEB) and custom forward/reverse primers (IDT). ATAC sequencing was carried out on an Illumina HiSeq X system (read length of 125 bp, paired-end).

### Bioinformatic analysis of ATAC sequencing data

ATAC sequencing reads were mapped to the GRCh38 reference genome and peaks were called following the processing pipeline developed by the ENCODE consortium (key resources information).

### Enhancer-gene map construction

An enhancer-gene map was estimated using the ABC model (version 0.2) (key resources information) with default parameters. The following files were used as inputs for the model: ATAC sequencing bam files for stimulated or non-stimulated SFs (prepared in this study), ChIP sequencing bam files for H3K27ac in stimulated or non-stimulated SFs (prepared in our previous study [26]), Hi-C contact map of stimulated or non-stimulated SFs (prepared in our previous study [26]), and gene expression data in stimulated or non-stimulated SFs (prepared in this study).

GWAS top variants for RA were downloaded from the GWAS catalog [27] on 22 October 2021. The major histocompatibility complex (MHC) region was excluded from the analysis. For each top variant, variants in LD (*r*^2^ > 0.8 in the European population) were tested for overlap with enhancers identified in the ABC model. For the enrichment estimation, variants were pruned and those in LD (*r*^2^ > 0.1) were regarded to be in the same loci.

### Stratified linkage disequilibrium score regression analysis

Stratified linkage disequilibrium score regression (S-LDSC) analysis was performed using LDSC software (version 1.0.1) (key resources information) for testing the association of genome-wide treatment effects with polygenic risks of common diseases. For this analysis, we used the top 15,000 downregulated open chromatin regions after each treatment. We adjusted for the enrichment of all accessible peaks in stimulated SFs (24 h after the combinatory stimulation with three types of cytokines without adding any agents) and the baseline model provided by the developers. For RA, enrichment analysis results from two GWAS (East Asian and European populations) were meta-analyzed with the inverse variance weighting method using normalized coefficients and their S.E. *P* values were calculated by testing whether the regression coefficient was significantly positive.

### Luciferase reporter assay

To assess the effect of four variants associated with *TRAF1* (rs2109896, rs7021049, rs7037195, and rs7021216) and one variant with *CD40* (rs6074022) on its potential enhancer activity, we used the Dual-Luciferase Reporter Assay system (Promega). For *TRAF1-*associated SNPs, 1711 bp fragments (hg19, Chr9: 123,682,842–123,684,552) containing different alleles of variants were cloned into the pGL4.26 vector: (1) **Protective**: rs2109896-T- rs7021049-T- rs7037195-C- rs7021216-A;(2) **rs2109896-Risk**: rs2109896-**C**- rs7021049-T- rs7037195-C- rs7021216-A;(3) **rs7021049-Risk**: rs2109896-T- rs7021049-**G**- rs7037195-C- rs7021216-A;(4) **rs7037195-Risk**: rs2109896-T- rs7021049-T- rs7037195-**T**- rs7021216-A;(5) **rs7021216-Risk**: rs2109896-T- rs7021049-T- rs7037195-C- rs7021216-**G**. For CD40-associated SNPs, 897 bp fragments (hg19, Chr20: 44,739,504–44740400) were used: (1) **rs6074022 Protective**: rs6074022-C;(2) **rs6074022 Risk**: rs6074022-**T**. All the constructs were verified by direct sequencing.

The HT-1080 cells were seeded to a 96-well plate with a density of 1 × 10^4^ per well 12 h before transfection and were co-transfected with the empty pGL4.26 or pGL4.26 vectors containing risk/protective alleles, along with an internal control pGL4.74 vector expressing Renilla luciferase (as a control for transfection efficiency) using Lipofectamine LTX with PLUS Reagent (Thermo Fishers) as per the manufacturer’s instructions. Twenty-four hours after transfection, cells were lysed and assayed for luciferase activity using a GloMAX Navigator System (Promega). Each experiment was performed in three replicates and was repeated at least three times.

### Knockdown assay

We used the CRISPR/Cas9 system to delete a small region surrounding rs7021049 or rs6074022. Streptococcus pyogenes Cas9 enzyme (Cas9 2NLS) and sgRNAs for each SNP were generated by Synthego. The complete sequences for all sgRNA templates are provided in Supplementary Table 2. Cas9 enzyme was mixed with sgRNAs in 1:1.3 ratios, and the resulting mixture was incubated for 10 min at room temperature to allow ribonucleoprotein (RNP) formation. For the MH7A cells, we used the Lipofectamine CRISPRMAX Reagent (Thermo Fishers) to deliver the RNP (ribonucleoprotein) complex as per the manufacturer’s instructions. Forty-eight hours after transfection, a mixture of three cytokines (100 U/mL IFN-γ, 5 ng/mL TNF-α, and 5 ng/mL IL-1β) was added, and the cells were stimulated for an additional 24 h. Subsequently, the cells were harvested and subjected to RNA isolation and real-time PCR (qRT-PCR) for *TRAF1* or *CD40*. Total RNA was extracted with the RNeasy Micro Kit (Qiagen) and was reverse-transcribed to cDNA with random primers (Invitrogen), dNTP mixture (Takara), ribonuclease inhibitor (Promega), and SuperScript III (Invitrogen). qRT-PCR was performed on the CFX Connect Real-Time PCR Detection System (Bio-Rad) using the QuantiTect SYBR Green PCR Kit (Qiagen). The primer pairs used in this study are shown in Supplementary Table 3. The relative expression was calculated based on the abundance of the control *GAPDH*. Each experiment was performed three times. The editing outcomes were measured with genomic PCR, Sanger sequencing, and analysis using ICE (https://ice.synthego.com/#/).

### Statistics

For in vitro analyses, statistical significance and analysis of variance (ANOVA) between indicated groups were analyzed by R (ver 3.4.1). A comparison of more than two group means was analyzed by Tukey’s multiple comparison tests. A comparison of two group means was analyzed by paired *t*-test. Statistically significant differences were accepted at *P* < 0.05 for all tests. Data in the bar charts were expressed as means ± standard deviation (SD). Multiple test correction was performed with the B-H method to obtain corrected *q*-values, unless otherwise explained.

## Results

### JAKis and the TNFi display distinctive transcriptomic signatures

**Fig. 1:**
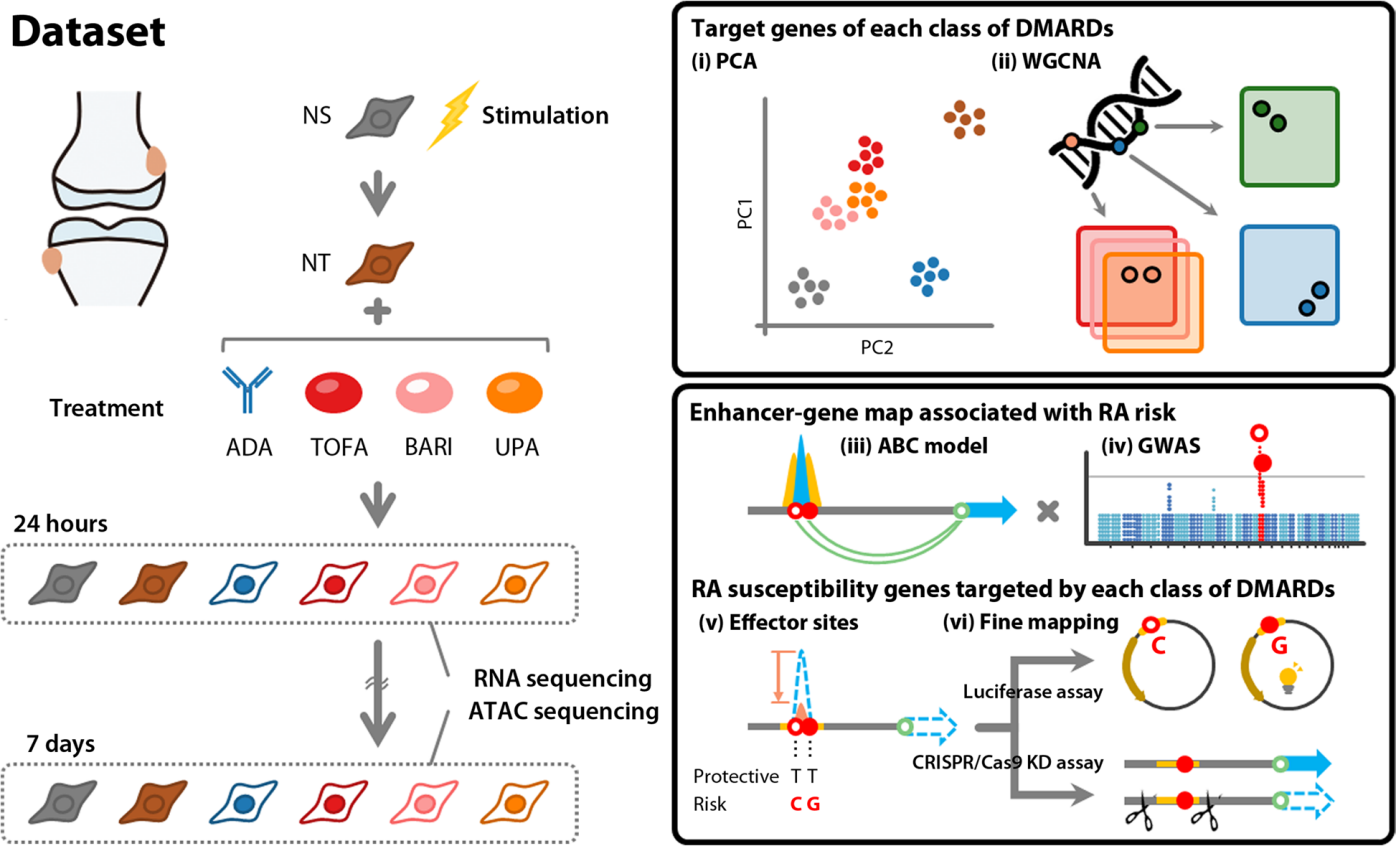
Experimental design for integrated analyses of action targets of therapeutic agents in synovial fibroblasts (SFs) from patients with rheumatoid arthritis (RA). Our study design included primary SFs from RA patients (*n* = 6) stimulated by a combination of three disease-relevant cytokines (10 ng/mL TNF-α, 10 ng/mL IL-1β, and 200 U/ml IFN-γ) and treated with three types of JAKis (4 µM tofacitinib, 4 µM baricitinib, or 4 µM upadacitinib) or a TNFi (20 µg/ml adalimumab). RNA sequencing and ATAC sequencing of individual samples were carried out 24 h and 7 days after treatment. Integrated analyses proceeded in order from (i) to (vi). ABC activity-by-contact, ADA adalimumab, BARI baricitinib, DMARDs disease modifying anti rheumatic drugs, GWAS genome-wide association studies, NS non-stimulated, NT non-treated, PCA principal component analysis, TOFA tofacitinib, UPA, upadacitinib, WGCNA weighted gene correlation network analysis

We stimulated primary SFs from six patients with RA with a combination of three disease-relevant cytokines (TNF-α, IL-1β, and interferon [IFN]-γ) and treated these with three types of JAKis (tofacitinib, baricitinib or upadacitinib) or a TNFi (adalimumab). The landscape of chromatin accessibility and the associated genome-wide transcriptome were integratively analyzed at 24 h and 7 days after treatment (Fig. 1).

**Fig. 2:**
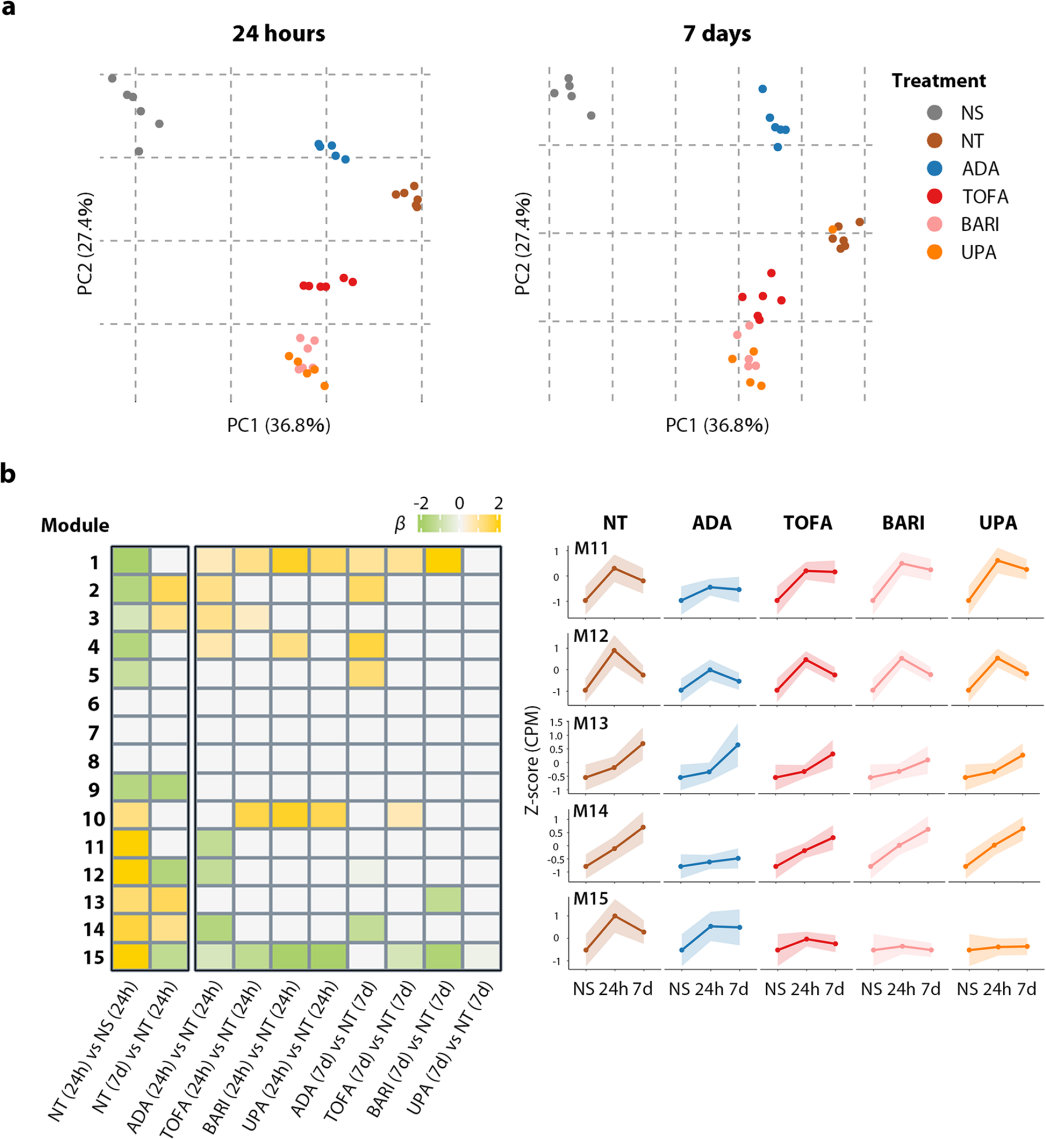
Distinctive transcriptomic signatures induced by therapeutic agents. **a** Principal component analysis (PCA) of gene expression levels for the top 1000 variable genes. Samples obtained at 24 h (left) and 7 days (right) after treatment were projected onto PC1/PC2. Numbers in parentheses indicate the contribution ratio (percentage of variation) of the first two PCs. **b** Heatmap illustrating the regression coefficient of the modules identified by weighted gene correlation network analysis (WGCNA) in comparing different treatment conditions (left). Temporal transcriptomic changes in representative modules are shown in line graphs (right). Dots in line plots represent the mean ADA adalimumab, BARI baricitinib, CPM count per million, M module, NS non-stimulated, NT non-treated, PC principal component, TOFA tofacitinib, UPA upadacitinib, 24 h 24 h, 7d 7 days

First, we assessed the trends in how each drug affects global gene expression (Fig. 1i). Principal component analysis (PCA) of transcriptomic signatures demonstrated that each treatment tends to revert activated SFs to their quiescent state as expected, and this effect was partially shared between JAKis and the TNFi (Fig. 2a, largely reflected as PC1). In contrast, inter-class differences between JAKis and the TNFi were confirmed 24 h after treatment and were largely preserved until 7 days after treatment (Fig. 2a, largely reflected as PC2). Overall, intra-class differences among JAKis were small (Supplementary Fig. 1). Thus, for simplicity, we mainly focused on the inter-class differences between JAKis and the TNFi on gene regulation for the following analyses.

**Fig. 3:**
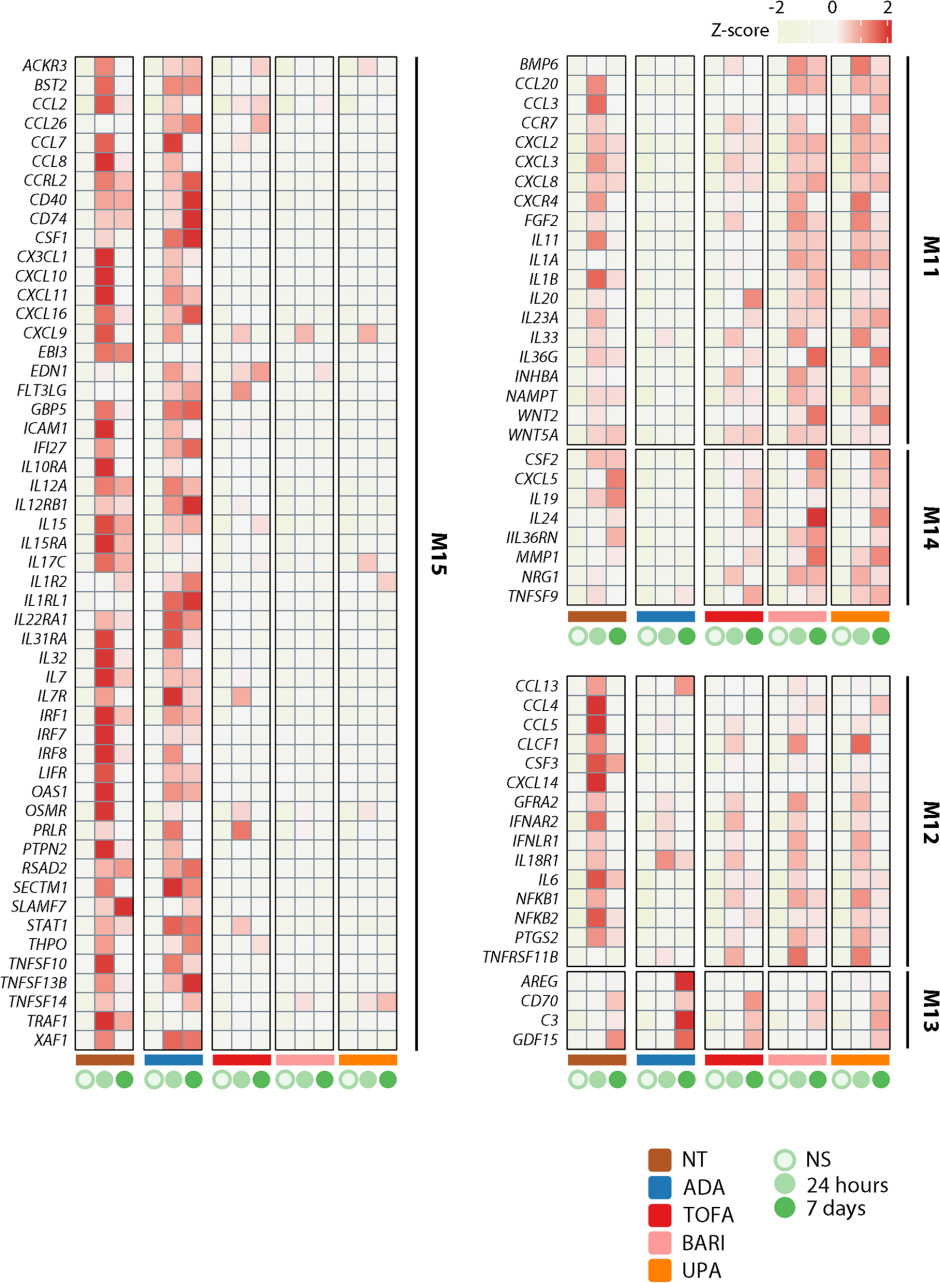
Inflammatory mediators targeted by JAKis and the TNFi. Temporal transcriptomic changes of inflammatory mediators in modules 11–15 are classified in Fig. 2b. Expression levels were Z normalized for each gene and the median value of the scores in each treatment condition is shown. ADA adalimumab, BARI baricitinib, M module, NS non-stimulated, NT non-treated, TOFA tofacitinib, UPA upadacitinib

**Fig. 4:**
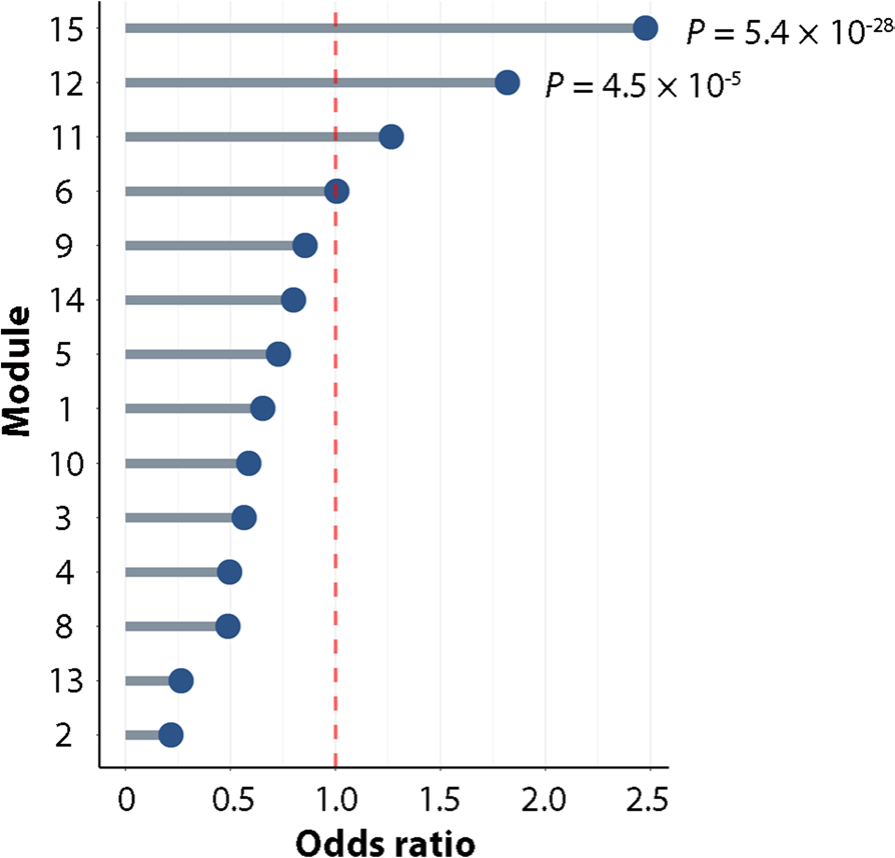
Transcriptomic signatures enriched in inflammatory super enhancer regions. Enrichment of modules classified in Fig. 2b in super-enhancers (SEs) of synovial fibroblasts (SFs) configured under a synergistic proinflammatory environment [26]. The red dashed line represents the cutoff values for Bonferroni significance

We next performed weighted gene correlation network analysis (WGCNA) to identify the gene networks that fluctuate under the influence of inflammation and therapeutic agents in a time-dependent manner (Fig. 1ii), and the detected genes were classified into 15 clusters (modules) by patterns of response to various perturbations (Fig. 2b). Genes of each WGCNA module are listed in Supplementary Table 4. Module 12 (M12), which contained various inflammatory mediators such as *IL6*, *NFKB1,* and *NFKB2,* was partially suppressed by both JAKis and the TNFi. In activated SFs under synergistic stimuli, the enhancer cluster (super-enhancer; SE) is composed upstream of *IL6*, and its proximity to the promoter region leads to an explosive increase in gene expression [26]. Despite the reduction of *IL6* expression under either treatment, this SE region retained the open chromatin structure (Supplementary Fig. 2). In contrast, module 11 (M11) and module 14 (M14), including *C-X-C motif chemokine receptor 4 (CXCR4)* and *colony-stimulating factor 2 (CSF2)* (Fig. 3), were representative gene clusters whose expression was enhanced under inflammatory stimuli and preferentially suppressed by the TNFi compared to JAKis for 7 days. On the other hand, module 15 (M15) was targeted more preferably by JAKis than the TNFi, and this cluster included *C–C motif chemokine ligand 8 (CCL8)* and *C-X-C motif chemokine ligand 10 (CXCL10)* (Fig. 3). We have previously reported that a synergistic proinflammatory environment, including TNF-α, IL-1β, and IFN-γ, induces dynamic chromatin structural rearrangement of SFs. RA risk loci accumulate in the resulting SEs, which is considered to be a critical region related to the disease heritability [26]. Since M15, M12, and M11 genes are significantly enriched in these inflammatory SE regions (Fig. 4), JAKis and the TNFi could exhibit shared and differential modification of these pathological epigenome structures.

As described for *IL6* in M12, M13 is another gene cluster highly expressed in response to inflammatory stimuli and was characterized by partial suppression, even with treatment with JAKis or the TNFi. *C3* is a typical example of a gene associated with M13, and this molecule has been reported to act on inflammatory tissue through metabolic reprogramming of SFs [28]. Importantly, the chromatin structure of the transcription start site (TSS) of the *C3* gene is opened by cytokine stimulation, and its epigenetic status remains unchanged after either treatment (Supplementary Fig. 3). Suppressing the complement system might be a novel target that current available therapeutic agents do not fully address.

### Putative enhancer-gene pairs analysis reveals tissue-specific disease susceptibility genes

**Fig. 5:**
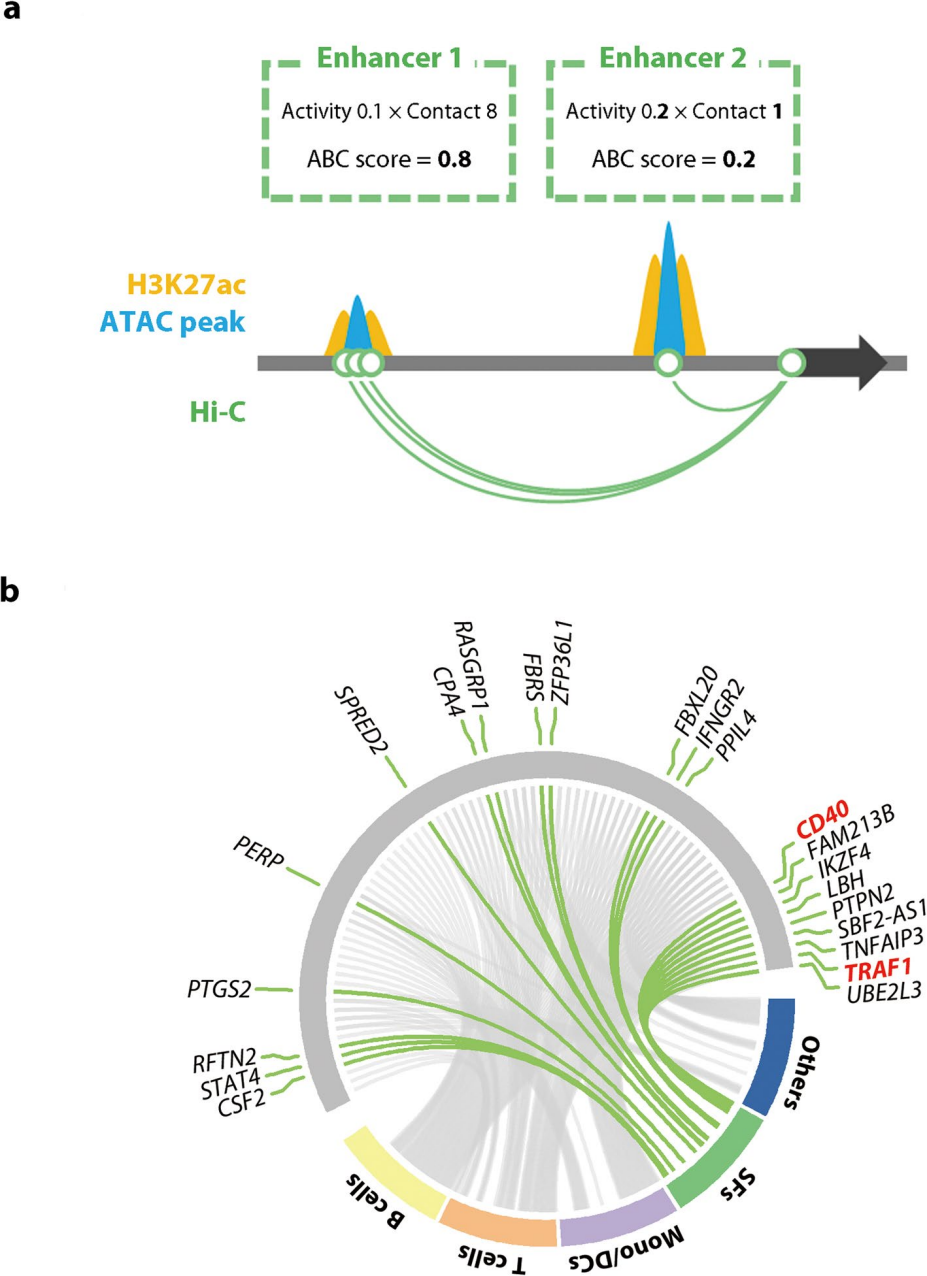
The overlapping of rheumatoid arthritis (RA) risk loci and the activity-by-contact (ABC) enhancers in various cell types. **a** A schematic image of the ABC model. **b** Circos plot showing the ABC enhancers overlapping with RA risk loci in various cell types, including B cells, T cells, monocytes/DCs, and synovial fibroblasts (SFs). ABC activity-by-contact, DCs dendritic cells

We next constructed an enhancer-gene map using the activity-by-contact (ABC) model [29] to elucidate the regulatory function of disease-associated genetic variants in activated SFs and the effects drugs have on these (Fig. 1iii). In this analytical pipeline, we combined the information of the open chromatin regions (defined with ATAC sequencing), active regulatory regions (defined with H3K27ac ChIP sequencing analysis), and 3D genome architectures (chromatin loops detected by Hi-C analysis) (Fig. 5a). All data was previously obtained from SFs [26].

As a result, 47,422 enhancer-gene pairs were estimated in SFs, and 10,561 pairs were specific for the activation state under synergistic stimuli. The enhancers identified by the ABC model predicted the gene expression much better than the open chromatin regions nearest to the TSS (Supplementary Fig. 5). In addition, the enhancer-gene connections of SFs defined in this study were closer to those of fibroblasts and osteoblasts than other cell types in public epigenome databases (roadmap epigenomics project [30] and ENCODE [31]) (Supplementary Fig. 6). Thus, these data suggested the validity of this analytical approach.

Next, we evaluated the enrichment of top variants from RA genome-wide association studies (GWAS) [27] in the ABC enhancers (Fig. 1iv). Among previously reported ABC links in > 100 tissues [32] together with those in our SFs (139 conditions in total), ABC enhancers were significantly enriched to RA GWAS top hits in 40 conditions (Supplementary Fig. 7). These included T cells, B cells, monocytes/dendritic cells (DCs), and activated SFs. In total, 81 enhancer-gene pairs showed significant overlap with the RA risk loci in at least one of these 40 conditions, among which 22 loci overlapped with activated SFs (Fig. 5b and Supplementary Fig. 8). Interestingly, a tissue-specific association was implied for some susceptibility genes. For instance, data suggested that the disease-relevant enhancers acted on *STAT4* and *CSF2* in SFs, but not in T cells or B cells.

### RA susceptibility regions are targeted differently by therapeutic agents

Interestingly, the chromatin structure of all ABC enhancers overlapped with RA risk loci opened upon synergistic stimuli, in contrast to bidirectional changes in the expression of their corresponding genes (Supplementary Fig. 9). This suggests that RA risk loci are enriched in the genomic region of SFs, where the influence is enhanced under inflammatory stimuli. It is presumed that gene expression tends to fluctuate under the influence of various secondary factors, whereas the epigenome is less susceptible to such factors.

We next sought to evaluate the direct action of JAKis and the TNFi on the regulatory machinery of genes associated with RA susceptibility (Fig. 1v). Firstly, to elucidate the effect of the TNFi, we integrated variation in gene expression with that of regulatory activity of the corresponding enhancer estimated by the ABC model (Supplementary Fig. 9). Among genes significantly modulated by the TNFi treatment, seven overlapped with disease-susceptibility genes in the analysis described in the previous section. Their corresponding ABC enhancers were all changed in the closing direction upon treatment. One example is *tumor necrosis factor receptor (TNFR) associated factor 1 (TRAF1)*, which plays a pivotal role in the immune system as an adapter protein to mediate intracellular signaling cascades [33, 34]. An open chromatin peak located on the intron of the *TRAF1* gene overlapped with RA risk SNPs (i.e., rs2109896, rs7021049, rs7021206, and rs7037195) and was suggested to be connected to the promoter of the gene in the ABC model (Supplementary Fig. 5a). The accessibility of this locus was the most significantly modified by the TNFi treatment in a genome-wide comparison. Interestingly, this locus has also been reported to be associated with treatment resistance to TNFis in a previous GWAS study [35–37]. These findings indicate that the TNFi exerts its efficacy by direct targeting of the *TRAF1* region, which is involved in the development and therapeutic responsiveness of RA.

**Fig. 6:**
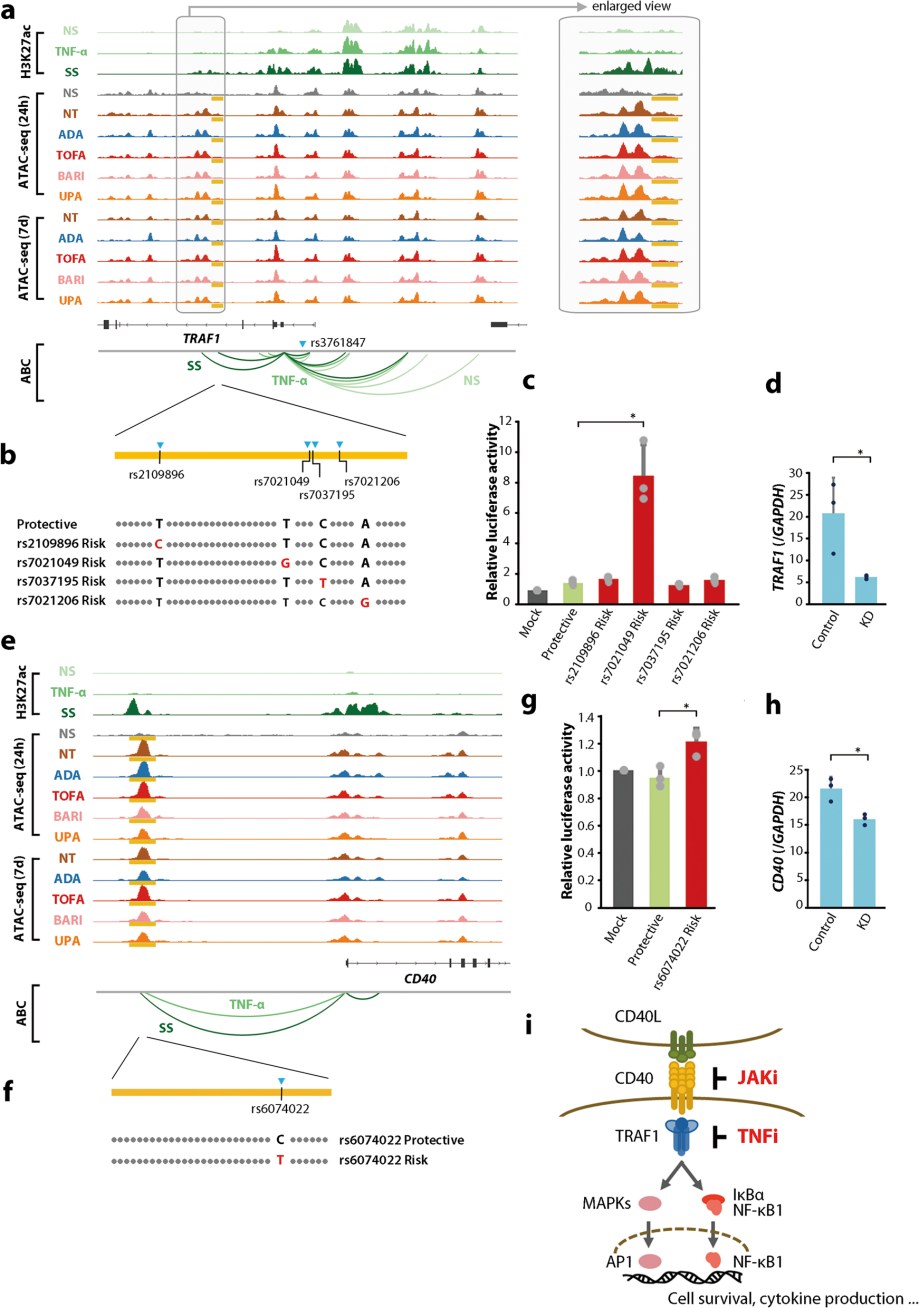
The direct actions of JAKis and the TNFi on the regulatory machinery of rheumatoid arthritis (RA) susceptibility genes. **a, e** Organization of transcriptional regulatory regions around the *TRAF1* (**a**) and *CD40* (**e**) gene. The boxed area indicates a putative enhancer that overlaps with RA risk SNPs. SS (synergistic stimuli) means a combination of eight different cytokines (IFN-α, IFN-γ, TNF-α, IL-1β, IL-6/sIL-6R, IL-17, TGF-β1, and IL-18) [26]. Data were visualized using the Integrative Genomics Viewer (IGV). **b, f** A schematic image of insertions into luciferase reporter vectors. **c, g** Relative luciferase activity of an allele-specific reporter assay (*n* = 3) using HT-1080 cells. Bars, mean; error bars, SD. *P* values were determined using paired *t*-test (*P < 0.05). **d, h** Transcript abundances of *TRAF1* (**d**) and *CD40* (**h**) obtained from qRT-PCR data in genetically edited cells (*n* = 3). A small region surrounding rs7021049 or rs6074022 was deleted in MH7A cells using the CRISPR/Cas9 system. Bars, mean; error bars, SD. *P* values were determined using paired *t*-test (*P < 0.05). **i** A graphical summary of the effector sites of JAKis and the TNFi on the CD40-TRAF1 cascade. ABC activity-by-contact, ADA adalimumab, BARI baricitinib, NS non-stimulated, NT non-treated, SS synergistic stimuli, TOFA tofacitinib, UPA upadacitinib, 24 h 24 h, 7d 7 days

The ABC model predicted the target genes of enhancers, but it does not necessarily mean that genetic variants affect enhancer activity or gene expression. Also, four SNPs exist in tight linkage disequilibrium (LD) in the *TRAF1* locus (Fig. 6a), which hampered the fine-mapping of the causal variant (Fig. 1vi). To experimentally map the causal variant in this region, we carried out luciferase reporter assays using the HT-1080 cell line and assessed the allele-specific regulatory potential of the 1711-bp sequence using a minimal promoter plasmid (Fig. 6b). We found marked enhancer activity with the rs7021049 risk allele (G) compared to the protective allele (T) (*P* < 0.05), and the other three SNPs (rs2109896, rs7021206, and rs7037195) showed no significant allelic effects (Fig. 6c). The transcriptional impact of the rs7021049 region was confirmed by clustered regularly interspaced short palindromic repeat (CRISPR)-based genome editing; *TRAF1* mRNA expression was significantly reduced in the MH7A cell lines in which the rs7021049 region was knocked down (Fig. 6d). These observations indicate that rs7021049 has critical enhancing effects on the *TRAF1* gene.

Next, we assessed the action targets of JAKis. As observed with the TNFi, the chromatin structures of 13 ABC enhancers that overlap with RA risk loci were all changed in the closing direction (Supplementary Fig. 9). One example is *CD40*, which is a member of the TNFR family, and interaction between CD40 and its ligand leads to various immunological responses, including B cell and T cell activation, upregulation of cell surface molecules relevant to inflammatory responses, and expression of cytokines [38]. An open chromatin peak located upstream of *CD40* and in contact with the promoter of the gene under synergistic stimuli overlapped with rs6074022, which is in tight LD (*r*^2^ = 0.9 in the East Asian, *r*^2^ = 0.95 in the European population) with an established RA risk SNP rs4810485 (Fig. 6e). JAKis downregulated the expression of the *CD40* gene together with a modification of this open chromatin region, while the TNFi did not affect it (Fig. 6e and Supplementary Fig. 9). We experimentally validated the allele-specific regulatory effects of rs6074022 on the *CD40* gene (Fig. 6f–h).

Consequently, these results provided clear evidence that the RA risk loci obtained from GWAS are direct targets for JAKis and the TNFi at different action points. It should be noted that epigenome modification of the risk site might serve as a novel therapeutic target when considering genomics-driven drug discovery. The relationship between the sites of action of these therapeutic agents and susceptibility loci of the various immune and inflammatory diseases is discussed in Supplemental Note.

## Discussion

In this study, we described the genetic, epigenetic, and immunological landscape of action targets of JAKis and the TNFi in activated SFs. Although our analyses were performed in vitro, these responses form the basis of clinical efficacy. JAKis induced distinct transcriptomic and epigenomic features on RA SFs compared to the TNFi, and enrichment of JAKis targeted modules in SEs of activated SFs is consistent with the clinical benefits of JAKis in a broad range of patients, including those with multidrug resistance. Moreover, the different inflammatory mediator profiles suppressed by JAKis and the TNFi is an interesting finding. Our results indicated that JAKis suppress gene expression of not only cytokines and their receptors but also intracellular signaling molecules such as *STAT1*, *IRF1*, and *IRF7*.

Importantly, we showed that JAKis and the TNFi modify genetic risk regions of RA differently. Although the understanding of human genetics of immune-mediated diseases has enhanced with recent large-scale GWAS, functional interpretation of these variants remains a major challenge. Considering GWAS variants are concentrated within active regulatory regions in disease-relevant cell types [39–45], we previously conducted cis-eQTL (expression quantitative trait locus) analysis and GWAS-SEs enrichment analysis of activated SFs and reported the promising link between chromatin remodeling of SFs under synergistic stimuli with the development of RA [26]. In this study, by constructing an enhancer-gene map using the ABC model, we clarified the targets of JAKis and the TNFi on the genetic risk of RA borne by activated SFs. One example of the TNFi targeted enhancer-gene pairs was the association of an enhancer overlapping with RA risk SNPs (i.e., rs2109896, rs7021049, rs7021206, and rs7037195) and the *TRAF1* promoter. TRAF1 is known as a bidirectional adaptor protein of NF-κB signaling, acting stimulative downstream of TNFRs and inhibitory downstream of Toll-like receptors [34]. A previous report showed the protein-level QTL association of TRAF1 in lipopolysaccharide (LPS)-stimulated monocytes and rs3761847, an established RA risk SNP in the *TRAF1* 5’ intronic region [46]. In this report, activated monocytes from healthy human subjects with the rs3761847 risk genotype (G) expressed less TRAF1 protein, but larger amounts of inflammatory cytokines in response to LPS. On the contrary, a larger eQTL study from our laboratory indicates that the direction of *TRAF1* expression regulation by this locus differs depending on the cell type [47]. For instance, in plasmacytoid dendritic cells, a genome-wide significant eQTL effect was found in the direction of upregulation of *TRAF1* expression when rs3761847 is a risk genotype (*P* = 2.1 × 10^–9^). In SFs, the rs3761847 region has a condensed chromatin structure even in the activated state, and it is less likely that this locus is functionally involved in *TRAF1* expression. Meanwhile, we identified different RA risk loci in activated SFs, and successfully fine-mapped rs7021049 as a causal variant in fibroblasts by in vitro assays. Furthermore, we showed that this region is the direct point of action of the TNFi.

In contrast, the ABC enhancer for *CD40* was an example of JAKis targets that overlapped with an RA risk SNP, rs6074022. As we previously reported, rs6074022 had robust eQTL effects on *CD40* in SFs, especially under a proinflammatory environment containing IFN-γ [26], and activation of CD40-CD40L signaling has also been demonstrated to promote expression of *IL6*, which is a crucial factor for osteoclast differentiation, and chemokines (e.g., *CCL5, CXCL10*) by SFs. In the thyroid-associated ophthalmopathy mouse model, the most common autoimmune diseases of the orbit, CD40 inhibitor, which specifically binds CD40-positive orbital fibroblasts inhibited TGF-β-induced cell viability and ameliorated inflammatory infiltration and the hyperplasia of orbital tissues [48]. Importantly, CD40 is known as one of the upstream inducers of TRAF1 signaling, and the CD40-mediated TRAF1 cascade activates the production of matrix metalloproteases and decreases apoptosis through the JNK and NF-κB pathways in RA SFs [49]. Furthermore, TRAF1 itself belongs to an NF-κB-dependent gene product [50], and triggering of CD40 results in the transcription of the *TRAF1* gene to form a positive feedback loop [51]. Taken together, JAKis and the TNFi inhibit this CD40-TRAF1 signaling, which could contribute to osteochondral destruction, synovial proliferation, and chronic inflammation caused by immune cell infiltration, and JAKi is thought to inhibit the upstream. In some RA patients who are highly dependent on this cascade, JAKis could be beneficial than TNFis, and more than that, the efficacy of JAKis might be enhanced in patients with rs6074022 risk polymorphisms. (Fig. 6i).

As mentioned above, the CD40 cascade regulates *IL6* expression [26]. Our epigenetic analysis indicated that both JAKis and the TNFi slightly alter the open chromatin structures of the *IL6* gene region induced by synergistic stimuli. These molecular targeted therapies might inhibit the function of genes regulating *IL6* expression, including CD40 and TRAF1, whereas *IL6* gene expression readiness remains untouched. The maintained open chromatin regions around the *IL6* gene region might be associated with partial suppression of *IL6* expression and limited achievement of drug-free remission by currently used molecular-targeted therapies.

There are some limitations to this study. First, the concentrations of cytokines and drugs added to SFs in the in vitro assays might not necessarily reflect the local joint environment in vivo. Second, previous reports have shown that culture procedures may affect the phenotype of SFs [52]. It should be noted that the results presented were obtained from early passage SFs, and using directly isolated cells might have led to a less biased analysis. Third, by comparing multiple JAKis concentration conditions, differences in JAK selectivity and off-target effects might become more apparent.

## Conclusions

Overall, our multilayered approach using drug-treated cells established a more detailed landscape of the genetic, epigenetic, and immunological action targets of current therapeutic agents. These findings are expected to serve as a foundation for clinical positioning of approved interventions in RA patients and may provide further opportunity for precision medicine.

### Supplementary Information

Additional file 1: Key resources information. **Supplementary Fig. 1** Overview of transcriptomic signatures in synovial fibroblasts (SFs) from patients with rheumatoid arthritis (RA) treated with therapeutic agents, related to Fig. 2 and 3. **Supplementary Fig. 2** Open chromatin structure of the *IL6* region remaining after treatment, related to Fig. 2 and 3. **Supplementary Fig. 3** The regulatory machinery of the *C3* gene that is less susceptible to modification by therapeutic drugs, related to Fig. 2 and 3. **Supplementary Fig. 4** Enhancer-gene pairs estimated by the activity-by-contact (ABC) model, related to Fig. 5 and 6. **Supplementary Fig. 5** Transcriptomic and epigenetic perturbation in different enhancer-gene pairing methods, related to Fig. 5 and 6.** Supplementary Fig. 6** Sharing of enhancer-gene connections between different cell types, related to Fig. 5 and 6. **Supplementary Fig. 7** Enrichment of rheumatoid arthritis (RA) GWAS top hits to enhancers identified by activity-by-contact (ABC) links in various tissues, related to Fig. 5 and 6. **Supplementary Fig. 8** Quantitative effect of the activity-by-contact (ABC) enhancers overlapping with the rheumatoid arthritis (RA) risk loci in various cell types, related to Fig. 5 and 6. **Supplementary Fig. 9** Overlap of rheumatoid arthritis (RA) risk loci for target regions of each therapeutic agent, related to Fig. 5 and 6. **Supplemental Note.** Heritability enrichment of immune and inflammatory diseases on the target sites of various therapeutics, related to Fig. 5 and 6. **Supplementary Table. 1 **Clinical background of RA SFs providers. **Supplementary Table. 2** Sequences of sgRNA templates used in the knockdown assay. **Supplementary Table. 3** Sequences of primer pairs used for qRT-PCR. **Supplementary Table. 4** Module information in WGCNA, related to Fig. 2 and 3.

## Data Availability

The datasets generated during this study are available at the National Bioscience Database Center (NBDC) with the study accession code E-GEAD-598.

## Abbreviations

ABC: Activity-by-contact
CCL8: C-C motif chemokine ligand 8
CRISPR: Clustered regularly interspaced short palindromic repeat
CSF2: Colony-stimulating factor 2
CXCL10: C-X-C motif chemokine ligand 10
CXCR4: C-X-C motif chemokine receptor 4
DC: Dendritic cell
DMARD: Disease-modifying anti-rheumatic drug
eQTL: Expression quantitative trait locus
GWAS: Genome-wide association studies
IGV: Integrative Genomics Viewer
IL: Interleukin
JAKi: Janus kinase inhibitor
LD: Linkage disequilibrium
LPS: Lipopolysaccharide
M: Module
NBDC: National Bioscience Database Center
PCA: Principal component analysis
RA: Rheumatoid arthritis
RCT: Randomized controlled trial
SE: Super-enhancer
SF: Synovial fibroblast
SNP: Single-nucleotide polymorphism
STAT: Signal transducer and activator of transcription
TNFi: Tumor necrosis factor-α inhibitor
TRAF1: Tumor necrosis factor receptor associated factor 1
TSS: Transcription start site
TYK2: Tyrosine kinase 2
WGCNA: Weighted gene correlation network analysis
