# Supplementary material for: Epigenetic targets of Janus kinase inhibitors are linked to genetic risks of rheumatoid arthritis
Source: Inflamm Regen. 2024 Jun 4;44:29. doi: 10.1186/s41232-024-00337-2 (PMC11149281; doi:10.1186/s41232-024-00337-2)
Supplement: Supplementary file 1 — Additional file 1: Key resources information. Supplementary Fig. 1 Overview of transcriptomic signatures in synovial fibroblasts (SFs) from patients with rheumatoid arthritis (RA) treated with therapeutic agents, related to Fig. 2 and 3. Supplementary Fig. 2 Open chromatin structure of the IL6 region remaining after treatment, related to Fig. 2 and 3. Supplementary Fig. 3 The regulatory machinery of the C3 gene that is less susceptible to modification by therapeutic drugs, related to Fig. 2 and 3. Supplementary Fig. 4 Enhancer-gene pairs estimated by the activity-by-contact (ABC) model, related to Fig. 5 and 6. Supplementary Fig. 5 Transcriptomic and epigenetic perturbation in different enhancer-gene pairing methods, related to Fig. 5 and 6. Supplementary Fig. 6 Sharing of enhancer-gene connections between different cell types, related to Fig. 5 and 6. Supplementary Fig. 7 Enrichment of rheumatoid arthritis (RA) GWAS top hits to enhancers identified by activity-by-contact (ABC) links in various tissues, related to Fig. 5 and 6. Supplementary Fig. 8 Quantitative effect of the activity-by-contact (ABC) enhancers overlapping with the rheumatoid arthritis (RA) risk loci in various cell types, related to Fig. 5 and 6. Supplementary Fig. 9 Overlap of rheumatoid arthritis (RA) risk loci for target regions of each therapeutic agent, related to Fig. 5 and 6. Supplemental Note. Heritability enrichment of immune and inflammatory diseases on the target sites of various therapeutics, related to Fig. 5 and 6. Supplementary Table. 1 Clinical background of RA SFs providers. Supplementary Table. 2 Sequences of sgRNA templates used in the knockdown assay. Supplementary Table. 3 Sequences of primer pairs used for qRT-PCR. Supplementary Table. 4 Module information in WGCNA, related to Fig. 2 and 3. [file 41232_2024_337_MOESM1_ESM.docx]

**Key resources information**

| **REAGENT or RESOURCE** | **SOURCE** | **IDENTIFIER** |
| --- | --- | --- |
| ***Chemicals, Peptides and Recombinant Proteins*** | | |
| Human IFN-γ | PeproTech | Cat#300-02; GenPept: P01579 |
| Human TNF-α | PeproTech | Cat#300-01A; GenPept: P01375 |
| Human IL-1β | PeproTech | Cat#200-01B; GenPept: P01584 |
| Adalimumab | AbbVie | N/A |
| Tofacitinib | Selleck | Cat#S2789;  CAS: 477600-75-2 |
| Baricitinib | Selleck | Cat#S2851;  CAS: 1187594-09-7 |
| Upadacitinib | AbbVie | N/A |
| ***Critical Commercial Assays*** | | |
| RNeasy Micro Kit | QIAGEN | Cat#74004 |
| TruSeq Stranded mRNA Library Prep Kit | Illumina | Cat#RS-122-2101/2102 |
| Tagment DNA TDE1 Enzyme and Buffer Kit | Illumina | Cat#20034197 |
| DNA Clean and Concentrator-5 Kit | Zymo | Cat#D4014 |
| Dual-Luciferase Reporter Assay system | Promega | Cat#E2920 |
| Lipofectamine LTX with PLUS Reagent | Thermo Fishers | Cat#15338100 |
| Lipofectamine CRISPRMAX Reagent | Thermo Fishers | Cat#CMAX00003 |
| ***Deposited Data*** | | |
| Read counts data of RNA sequencing | This paper | NBDC: E-GEAD-598 |
| Peaks data of ATAC sequencing | This paper | NBDC: E-GEAD-598 |
| ***Experimental Models: Cell Lines*** | | |
| Synovial fibroblasts of human rheumatoid arthritis | Articular Engineering | Cat#CDD-H-2910-RA |
| HT-1080 | Japanese Collection of Research Bioresources Cell Bank | JCRB9113 |
| MH7A | Riken cell bank | RCB1512 |
| ***Software and Algorithms*** | | |
| R3.4.1 | R Core team, 2017 | https://www.R-project.org |
| Cutadapt (version 1.16) | Martin et al, 2011 | https://github.com/marcelm/cutadapt |
| FASTX-Toolkit (version 0.0.14) | Gordon et al, 2010 | https://github.com/agordon/fastx_toolkit |
| STAR (version 2.5.3) | Dobin et al, 2014 | https://github.com/alexdobin/STAR |
| GENCODE (version 27) | Frankish et al, 2019 | https://www.gencodegenes.org/human/release_27.html |
| HTSeq (version 0.11.2) | Anders et al, 2010 | https://github.com/simon-anders/htseq |
| variancePartition | Hoffmann et al, 2021 | https://github.com/GabrielHoffman/variancePartition |
| Bowtie2 (version 2.3.4.2) | Langmead et al, 2013 | https://github.com/BenLangmead/bowtie2 |
| MACS 2.0 (version 2.1.1) | Zhang et al, 2011 | https://github.com/taoliu/MACS |
| PLINK (version 1.90b4.4) | Purcell et al, 2015 | https://github.com/chrchang/plink-ng/ |
| BEDTools (version 2.26.0) | Quinlan et al, 2013 | https://github.com/arq5x/bedtools2 |
| ABC model (version 0.2) | Fulco CP et al, 2019 | https://github.com/broadinstitute/ABC-Enhancer-Gene-Prediction |
| S-LDSC (version 1.0.1) | Finucane HK et al, 2015 | https://github.com/bulik/ldsc |
| ***Other*** | | |
| Digitonin | Promega | Cat#G9441 |
| Tween-20 | Sigma | Cat#11332465001 |
| NEBNext High-Fidelity 2X PCR Master Mix | NEB | Cat#M0541L |
| Custom Forward/Reverse Primer | IDT | N/A |
| SYBR Green I Nucleic Acid Gel Stain | Takara | Cat#5761A |
| SuperScript III Reverse Transcriptase | Invitrogen | Cat#18080093 |
| Random Primers | Invitrogen | Cat#48190011 |
| dNTP Mixture | Takara | Cat#4030 |
| RNasin® Plus Ribonuclease Inhibitor | Promega | Cat#N2611 |
| QuantiTect SYBR Green PCR Kit | QIAGEN | Cat#204143 |
| pGL4.26 vector | Promega | Cat#E8441 |
| pGL4.74 vector | Promega | Cat#E6921 |
| Cas9 2NLS Nuclease | Synthego | Cat#00033 |
| sgRNA (unmodified) kit | Synthego | N/A |

**
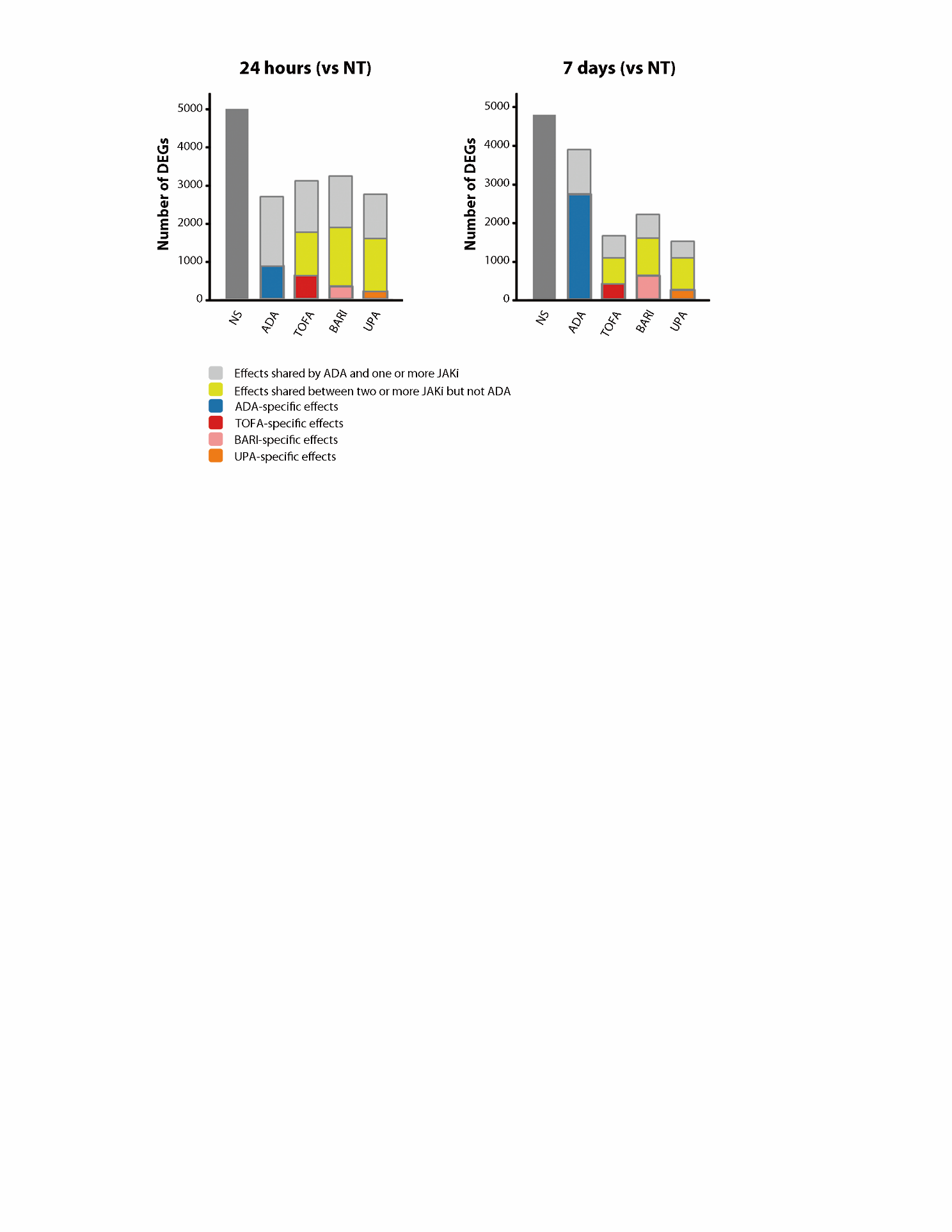
**

**Supplementary Fig. 1 Overview of transcriptomic signatures in synovial fibroblasts (SFs) from patients with rheumatoid arthritis (RA) treated with therapeutic agents, related to Fig. 2 and 3.**

The number of differentially expressed genes (DEGs) from RNA sequencing. Left, 24 hours after treatment (treated vs. non-treated); Right, 7 days after treatment (treated vs. non-treated). Bar plots are colored by effect sharing status.

*ADA, Adalimumab; BARI, Baricitinib; DEGs, differentially expressed genes; NS, non-stimulated; NT, non-treated; TOFA, Tofacitinib; UPA, Upadacitinib.*

**
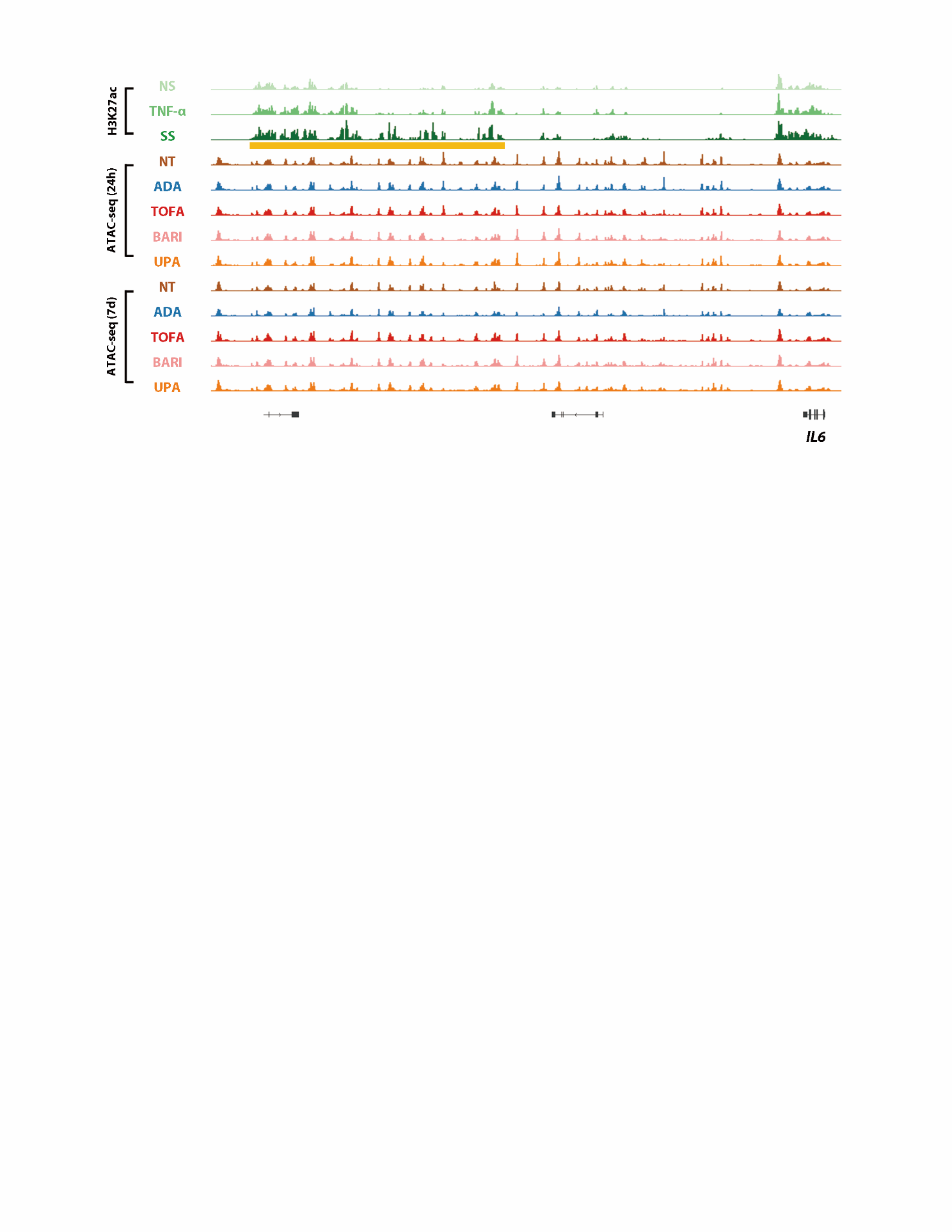
**

**Supplementary Fig. 2 Open chromatin structure of the *IL6* region remaining after treatment, related to Fig. 2 and 3.**

Organization of the transcriptional regulatory regions around the *IL6* gene. The yellow underlined region indicates super-enhancers (SEs) that are formed under inflammation and are sterically close to the *IL6* promoter. SS (synergistic stimuli) means a combination of eight different cytokines (IFN-α, IFN-γ, TNF-α, IL-1β, IL-6/sIL-6R, IL-17, TGF-β1, and IL-18) (1). Data were visualized using the Integrative Genomics Viewer (IGV).

*ADA, Adalimumab; BARI, Baricitinib; NS, non-stimulated; NT, non-treated; SS, synergistic stimuli; TOFA, Tofacitinib; UPA, Upadacitinib; 24h, 24 hours; 7d, 7 days.*

**
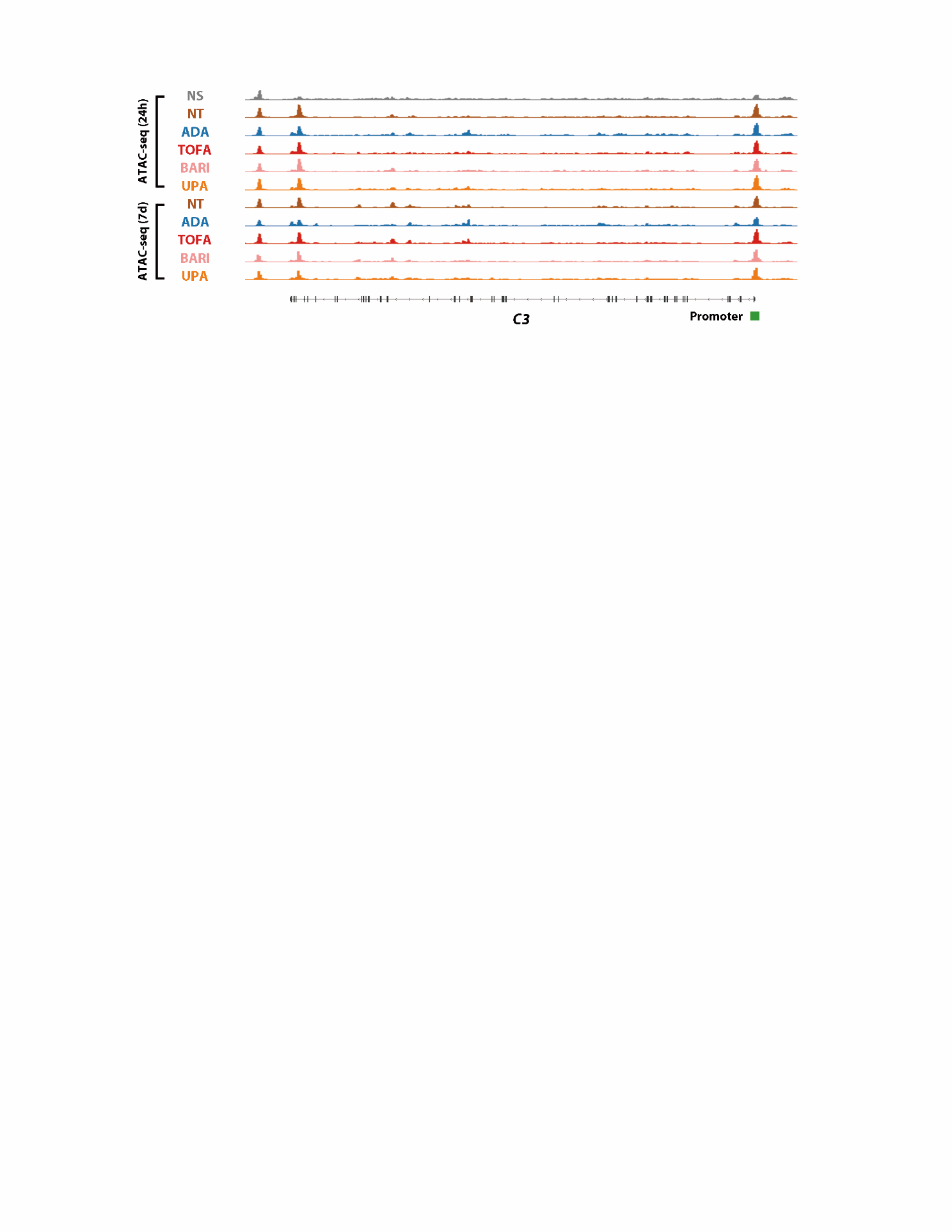
**

**Supplementary Fig. 3 The regulatory machinery of the *C3* gene** **that is less susceptible to modification by therapeutic drugs, related to Fig. 2 and 3.**

Organization of the transcriptional regulatory regions around the *C3* gene. The green box shows the promoter region. Data were visualized using the Integrative Genomics Viewer (IGV).

*ADA, Adalimumab; BARI, Baricitinib; NS, non-stimulated; NT, non-treated; SS, synergistic stimuli; TOFA, Tofacitinib; UPA, Upadacitinib; 24h, 24 hours; 7d, 7 days.*

**
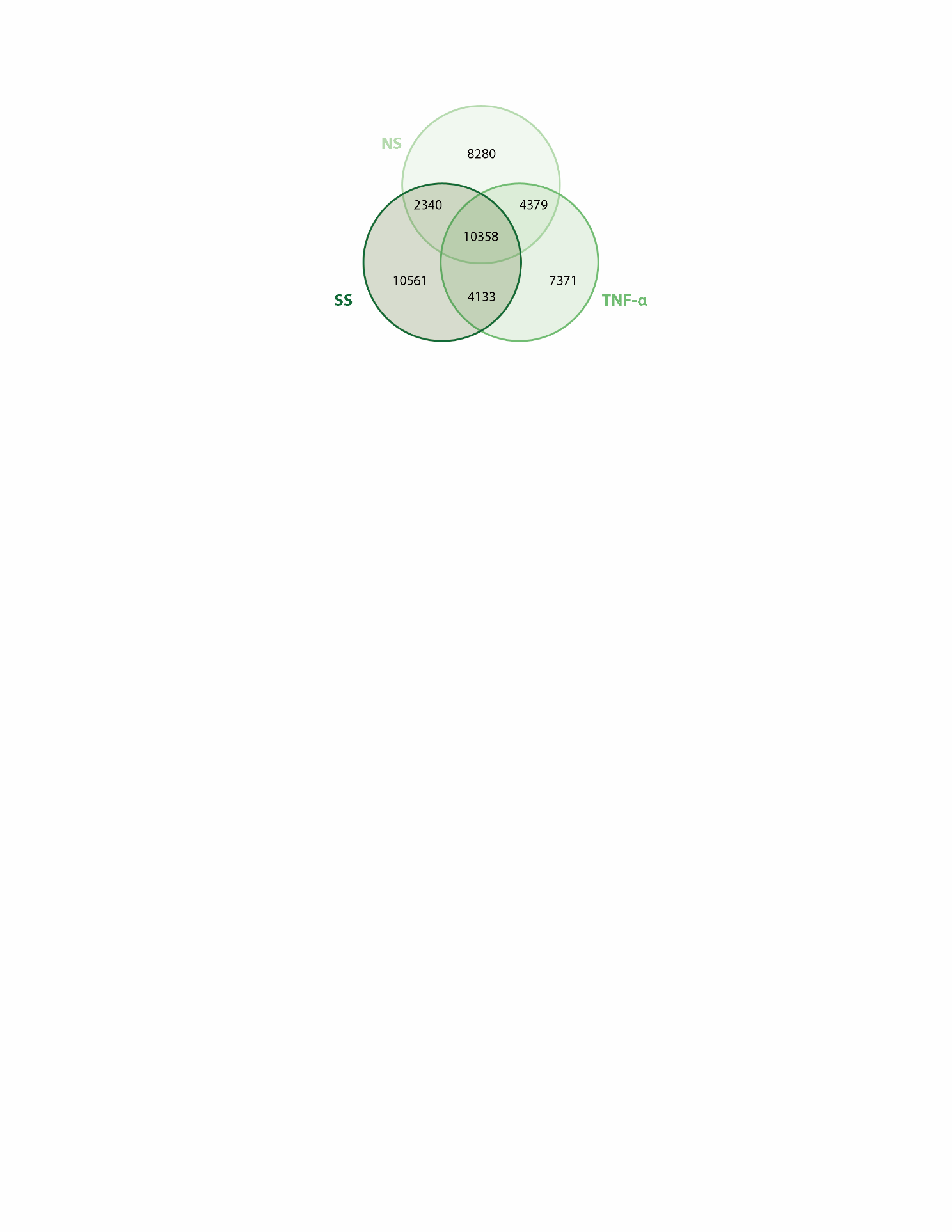
**

**Supplementary Fig. 4 Enhancer-gene pairs estimated by the activity-by-contact (ABC) model, related to Fig. 5 and 6.**

A Venn diagram representing the overlap of enhancer-gene pairs in synovial fibroblasts (SFs) under different stimulatory conditions.

*ABC, activity-by-contact; SS, synergistic stimuli.*

**
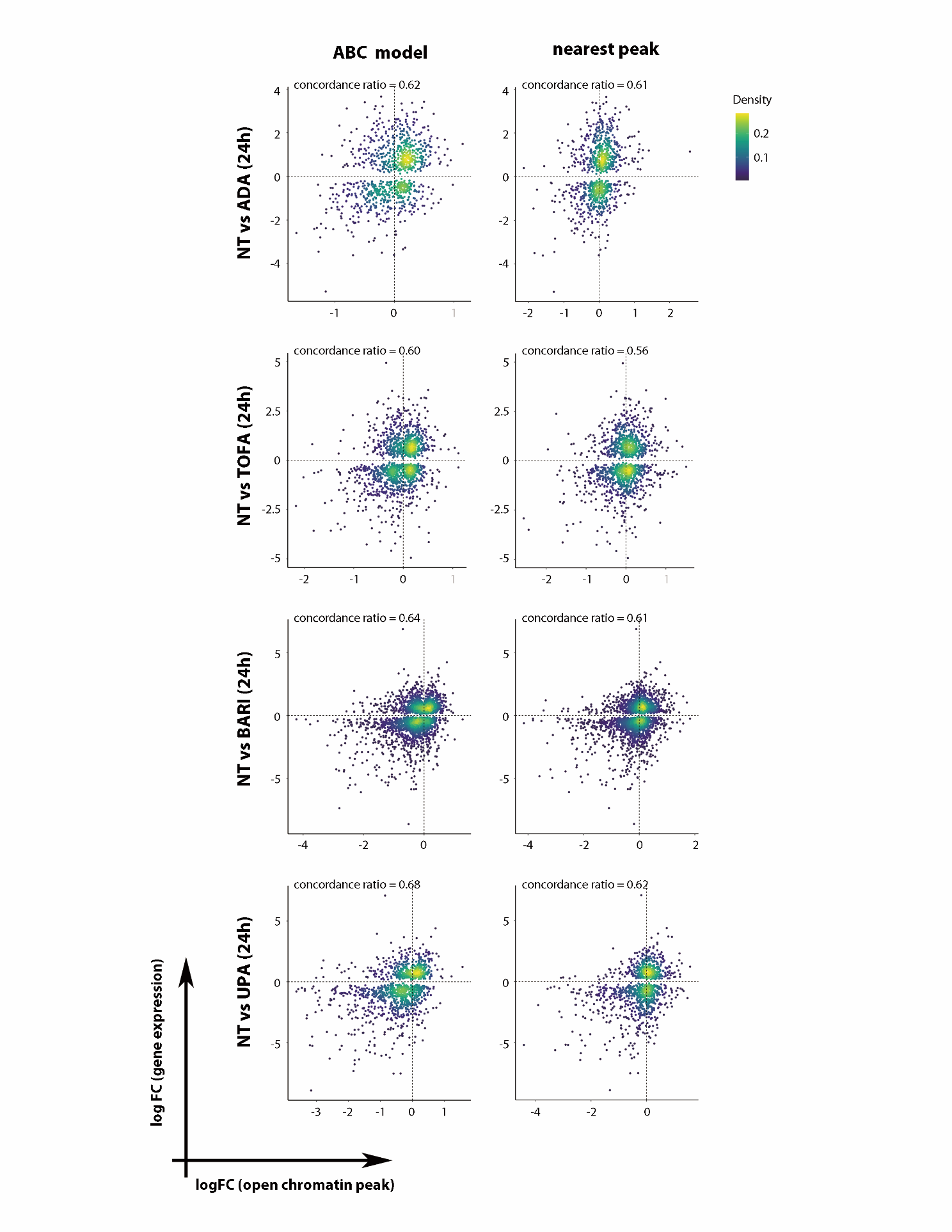
**

**Supplementary Fig. 5 Transcriptomic and epigenetic perturbation in different enhancer-gene pairing methods, related to Fig. 5 and 6.**

Scatter plots comparing variation in gene expression with that in regulatory activity of the corresponding enhancer estimated by the activity-by-contact (ABC) model or the nearest peak to the promoter.

*ABC, activity-by-contact; ADA, Adalimumab; BARI, Baricitinib; logFC, log fold change; NS, non-stimulated; NT, non-treated; TOFA, Tofacitinib; UPA, Upadacitinib; 24h, 24 hours.*

**
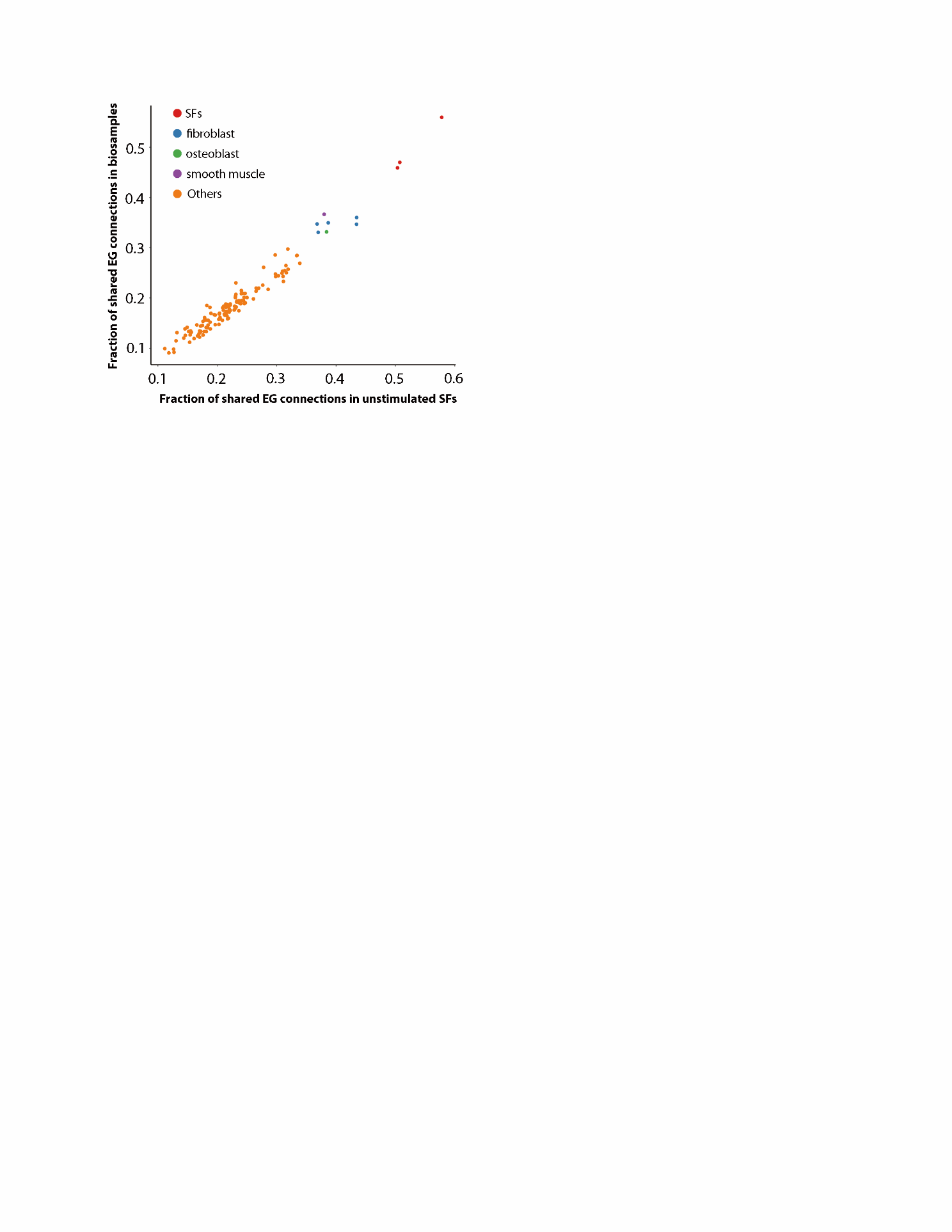
**

**Supplementary Fig. 6 Sharing of enhancer-gene connections between different cell types, related to Fig. 5 and 6.**

Scatter plots comparing shared enhancer-gene pairs estimated by the activity-by-contact (ABC) model between unstimulated synovial fibroblasts (SFs).

*ABC, activity-by-contact; EG, enhancer-gene.*

**
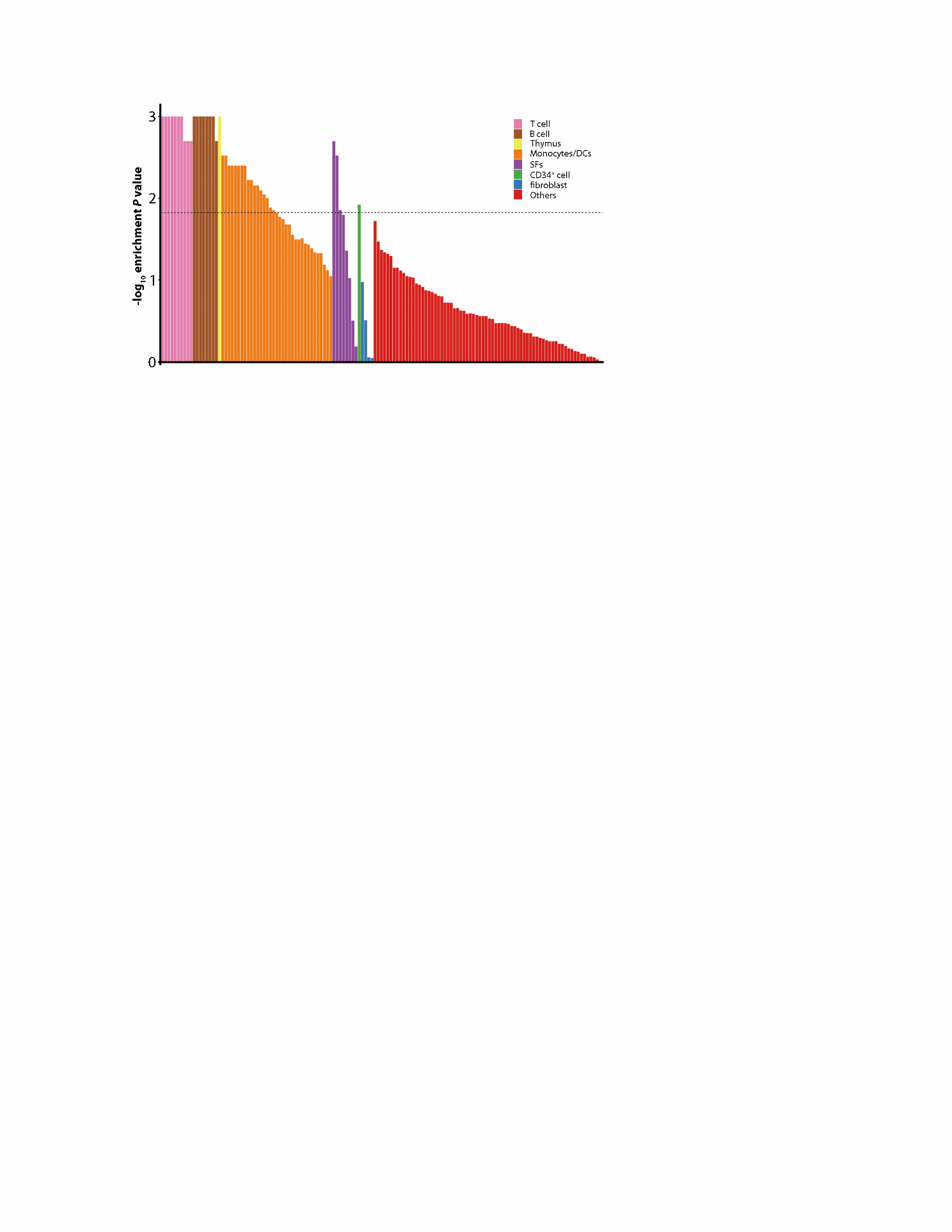
**

**Supplementary Fig. 7 Enrichment of rheumatoid arthritis (RA) GWAS top hits to enhancers identified by activity-by-contact (ABC) links in various tissues, related to Fig. 5 and 6.**

A bar chart representing enrichment *P* values calculated by permuting the GWAS top variants. The black dashed line corresponds to FDR = 0.05.

*ABC, activity-by-contact; DCs, dendritic cells; FDR, false discovery rate; GWAS, genome-wide association studies.*

**
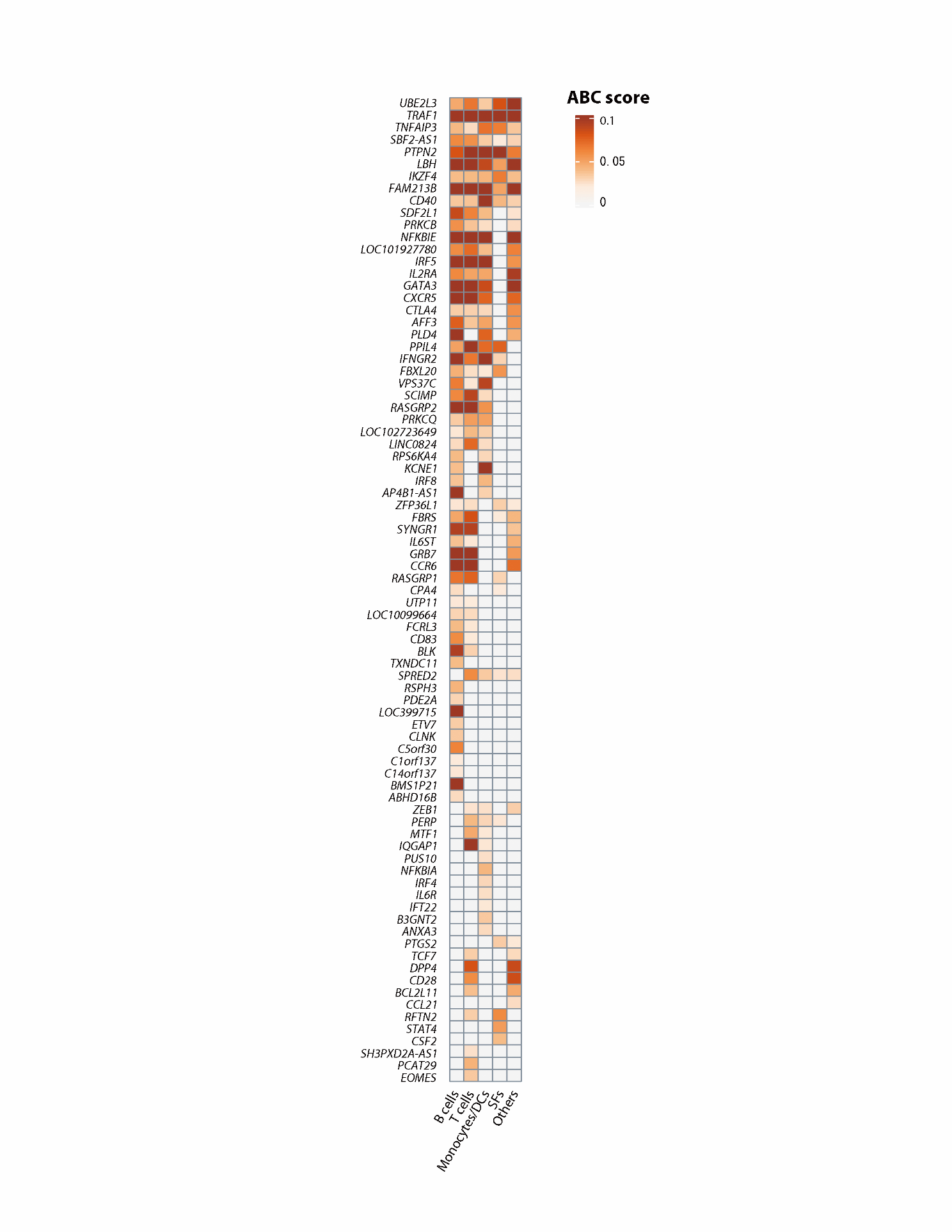
**

**Supplementary Fig. 8 Quantitative effect of the activity-by-contact (ABC) enhancers overlapping with the rheumatoid arthritis (RA) risk loci in various cell types, related to Fig. 5 and 6.**

Heatmap showing the ABC score of enhancers overlapping with RA risk loci in various cell types including B cells, T cells, monocytes/DCs, and synovial fibroblasts (SFs).

*ABC, activity-by-contact; DCs, dendritic cells.*

**
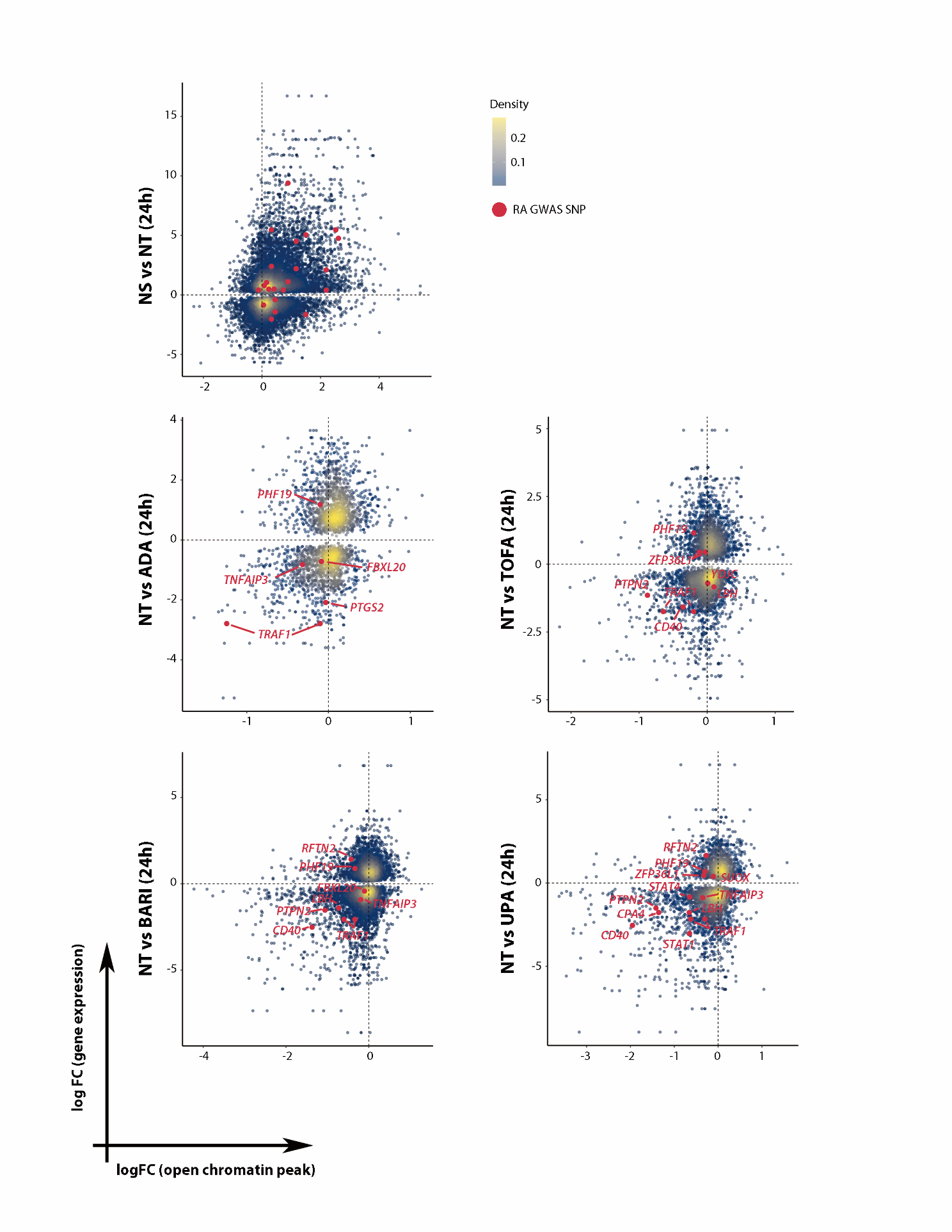
**

**Supplementary Fig. 9 Overlap of rheumatoid arthritis (RA) risk loci for target regions of each therapeutic agent, related to Fig. 5 and 6.**

Scatter plots representing the relationship of drug-modified enhancer-gene pairs and RA risk loci. Orange points indicate the activity-by-contact (ABC) enhancers overlapping with RA risk loci.

*ABC, activity-by-contact; ADA, Adalimumab; BARI, Baricitinib; GWAS, genome-wide association studies; logFC, log fold change; NS, non-stimulated; NT, non-treated; TOFA, Tofacitinib; UPA, Upadacitinib; 24h, 24 hours.*

**
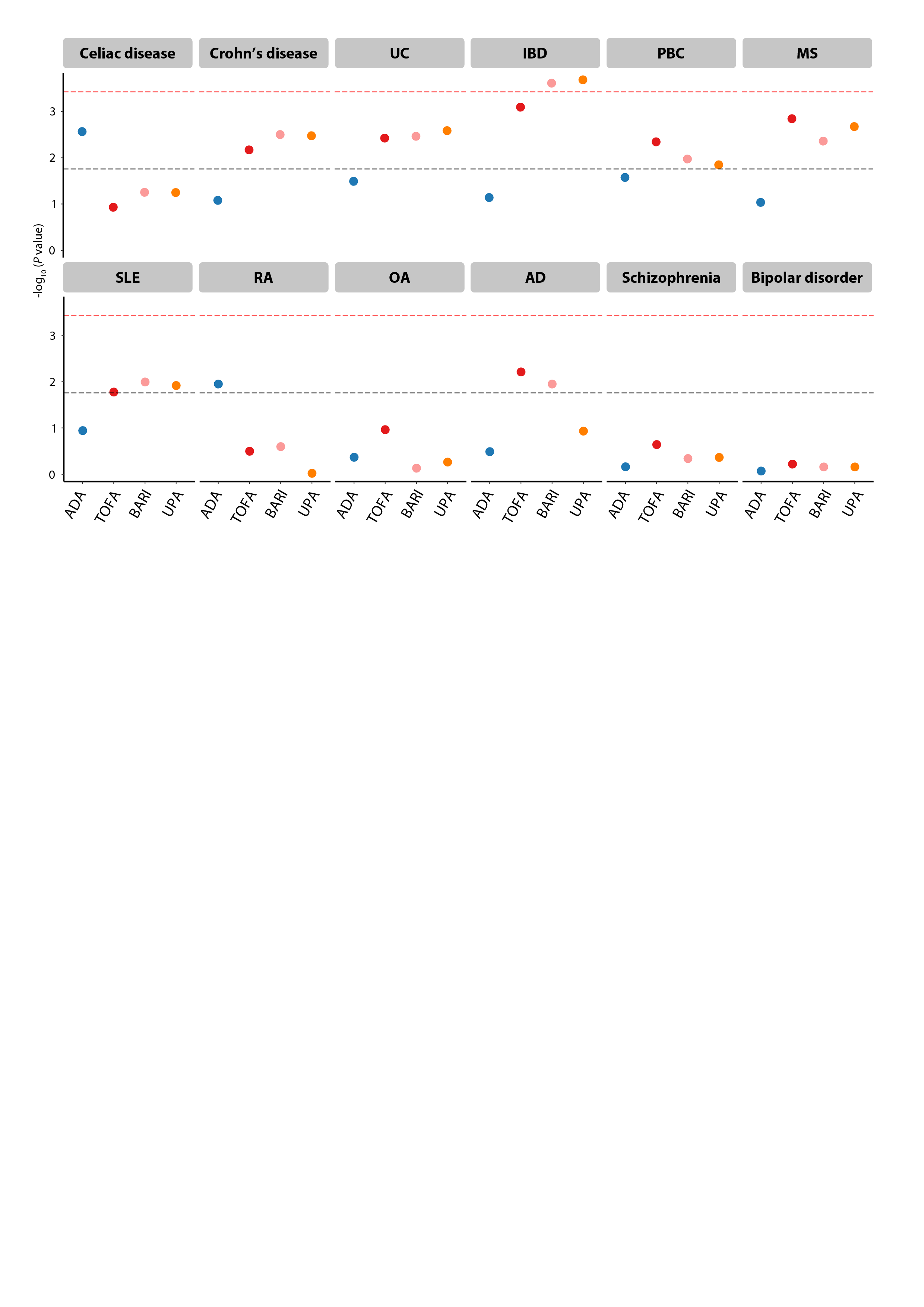
**

**Supplemental Note. Heritability enrichment of immune and inflammatory diseases on the target sites of various therapeutics, related to Fig. 5 and 6.**

The unique impact that some of the treatment targets of JAKis and the TNFi overlap with rheumatoid arthritis (RA) risk loci motivated us to verify whether such an effect is found in a genome-wide manner. To this end, we performed stratified linkage disequilibrium score regression (S-LDSC) (2, 3) using differential accessibility regions (DARs) in response to JAKis and the TNFi as an annotation. This showed that the target site of the TNFi significantly overlapped with RA heritability. Furthermore, by applying S-LDSC with the same annotation for GWAS of various immune and inflammatory diseases, it was demonstrated that the heritable risk of inflammatory bowel disease (IBD), multiple sclerosis (MS), and Alzheimer's disease overlapped with target sites of JAKis, and of Celiac’s disease with the TNFi. In fact, it has been reported that JAK-STAT signaling plays a significant role in the inflammatory pathogenesis of IBD (4) and tofacitinib has been approved in multiple countries for the treatment of patients with refractory ulcerative colitis (5). Collectively, these are thought-provoking results that have potential for future drug repositioning.

The red dashed lines and the black dashed lines are the cutoff values for Bonferroni significance and *P* = 0.05, respectively.

*AD, Alzheimer's disease; ADA, Adalimumab; BARI, Baricitinib; GWAS, genome-wide association studies;* *IBD, inflammatory bowel disease; logFC, log fold change; MS, multiple screlosis; NS, non-stimulated; NT, non-treated; OA, osteoarthritis;* *PBC, primary biliary cholangitis; SLE, systemic lupus erythematosus; TOFA, Tofacitinib; UC, ulcerative colitis; UPA, Upadacitinib.*

**Supplementary Table. 1 Clinical background of RA SFs providers.**

| **Cell line No.** | **Age** | **Race** | **Cause of death** |
| --- | --- | --- | --- |
| 1  2  3  4  5  6 | 66  57  68  86  80  67 | Caucasian  Caucasian  Caucasian  Caucasian  Caucasian  Caucasian | Chronic obstructive pulmonary disease  Stomach cancer  Heart failure  Alzheimer's disease  Myocardial infarction  Traumatic brain injury |

**Supplementary Table. 2 Sequences of sgRNA templates used in the knockdown assay.**

| **Target** | **sgRNA Target Sequence (5’ to 3’)** |
| --- | --- |
| TRAF1-1  TRAF1-2  TRAF1-3  CD40-1  CD40-2 | GAACTTTACACTCTGCAACG  TCAGCAACCTCTCCATCGGC  TGTGGACACTCGCCTGCCTC  CTCAGCACTCAGGCAGCCTA  GCAGACAGCTGCTTCCCCAG |

**Supplementary Table. 3 Sequences of primer pairs used for qRT-PCR.**

| **Target** | **Sequence (Forward)** | **Sequence (Reverse)** |
| --- | --- | --- |
| *TRAF1*  *CD40*  *GAPDH* | 5’- GGAAGCTGCGTGTGTTTGAG -3’  5’- GGTTTCTGATACCATCTGCGA -3’  5’- GAAGGTGAAGGTCGGAGTC -3’ | 5’- AGCTGGCTCTGGTGGATAGA -3’  5’- CTTTGGTCTCACAGCTTGTCC -3’  5’- GAAG ATGGTGATGGGATTTC -3’ |

**Supplementary Table. 4 Module information in WGCNA, related to Fig. 2 and 3.**

This table is uploaded separately.

**Supplemental References**

1. Tsuchiya H, Ota M, Sumitomo S, Ishigaki K, Suzuki A, Sakata T, et al. Parsing multiomics landscape of activated synovial fibroblasts highlights drug targets linked to genetic risk of rheumatoid arthritis. Ann Rheum Dis. 2021;80(4):440-50.

2. Finucane HK, Bulik-Sullivan B, Gusev A, Trynka G, Reshef Y, Loh PR, et al. Partitioning heritability by functional annotation using genome-wide association summary statistics. Nat Genet. 2015;47(11):1228-35.

3. Gazal S, Finucane HK, Furlotte NA, Loh PR, Palamara PF, Liu X, et al. Linkage disequilibrium-dependent architecture of human complex traits shows action of negative selection. Nat Genet. 2017;49(10):1421-7.

4. Salas A, Hernandez-Rocha C, Duijvestein M, Faubion W, McGovern D, Vermeire S, et al. JAK-STAT pathway targeting for the treatment of inflammatory bowel disease. Nat Rev Gastroenterol Hepatol. 2020;17(6):323-37.

5. Sandborn WJ, Su C, Sands BE, D'Haens GR, Vermeire S, Schreiber S, et al. Tofacitinib as Induction and Maintenance Therapy for Ulcerative Colitis. N Engl J Med. 2017;376(18):1723-36.
